# Molecular Mechanisms and Network Integration of Oxylipin Signaling in Plant Abiotic Stress Tolerance

**DOI:** 10.3390/ijms27188362

**Published:** 2026-09-19

**Authors:** Guljakhon Eshbekova, Anh Duc Tran, Kyoungwon Cho, Manh An Vu, Jeong-Il Kim, Hanh Thi Thuy Nguyen, Oksoo Han

**Affiliations:** 1 Graduate School and Kumho Life Science Laboratory, Department of Integrative Food, Bioscience and Biotechnology, College of Agriculture and Life Sciences, Chonnam National University, Gwangju 61186, Republic of Korea; chinboevaguljakhon@gmail.com (G.E.); anhduchytq1997@gmail.com (A.D.T.); kw.cho253@gmail.com (K.C.); manhanhd98@gmail.com (M.A.V.); kimji@jnu.ac.kr (J.-I.K.); 2 Molecular Biotechnology and Modern Botanical Research Laboratory, Department of Biotechnology and Plant Genomics, Samarkand State University named after Sharof Rashidov, University Boulevard 15, Samarkand 140100, Uzbekistan; 3 Faculty of Biotechnology, Vietnam National University of Agriculture, Hanoi 12406, Vietnam; 4 Department of Molecular Biotechnology, Chonnam National University, Gwangju 61186, Republic of Korea

**Keywords:** oxylipins, jasmonates, OPDA, oxylipin signaling, abiotic stress, signal crosstalk, ROS, calcium signaling, MAPK signaling, plant stress tolerance

## Abstract

Plant oxylipins are a diverse group of oxygenated fatty acid derivatives that function as important signaling molecules in plant responses to abiotic stress. Although jasmonates, particularly jasmonic acid (JA) and its derivatives, have been extensively studied, increasing evidence demonstrates that other oxylipin classes, including 12-oxophytodienoic acid (OPDA), green leaf volatiles (GLVs), reactive electrophilic oxylipins (RES), and peroxygenase (PXG)-derived oxylipins, also contribute to stress adaptation. This review summarizes current understanding of the molecular mechanisms underlying oxylipin signal perception, transduction, and regulation, with particular emphasis on interactions with other plant hormone pathways, reactive oxygen species (ROS), Calcium (Ca^2+^), and mitogen-activated protein kinase (MAPK) signaling. We further examine transcriptional, post-transcriptional, and post-translational mechanisms that regulate oxylipin responses and discuss their integration across individual and combined abiotic stresses. Particular attention is given to experimentally established mechanisms while distinguishing emerging or unresolved signaling processes. Understanding these interconnected signaling mechanisms will be important for developing strategies to improve crop resilience under global climate change.

## 1. Introduction

Agricultural productivity is facing serious challenges worldwide due to abiotic stress associated with global climate change. Environmental factors such as drought, salinity, heat and cold stress, heavy metal contamination and ultraviolet (UV) radiation negatively impact plant growth, development, and yield, resulting in economic losses globally [1,2,3]. Plants are often exposed to combinations of multiple abiotic stresses, which leads to severe damage [4]. Climate change is expected to increase extreme weather events, bringing serious consequences to agricultural ecosystems, threatening food security and crop production worldwide [5,6].

In recent years, there has been a lot of research into the mechanisms by which plants sense and respond to abiotic stressors at both the whole-body and individual cell levels. The perception and response to abiotic stresses in plants are complex processes involving numerous signaling components. Plant hormones traditionally play critical roles in regulating stress responses and adaptation mechanisms [7,8]. Abiotic stress factors induce membrane remodeling and lipid peroxidation, resulting in the release of polyunsaturated fatty acids (PUFAs). These serve as precursors for various bioactive signaling compounds [9,10]. These lipid-derived mediators are crucial components of stress signaling networks in plants, comprising phosphatidic acid, sphingolipids, phosphoinositides and oxylipins, participate in processes such as plant growth, development, defense, and adaptation to environmental stress [11,12,13]. Among them, oxylipins are biologically important molecules characterized by their structural and compositional diversity.

Oxylipins are oxygenated derivatives of PUFAs formed via enzymatic or non-enzymatic pathways. This group of substances includes oxygenated lipid metabolites such as jasmonic acid (JA), 12-oxophytodienoic acid (OPDA), green leaf volatiles (GLVs), and peroxygenase (PXG)-derived oxylipins, ketols, divinyl ethers, phytoprostanes (PhytoPs), phytofurans and complex esterified oxylipins [14,15,16,17,18,19]. Early studies on plant oxylipins focused on the lipoxygenase (LOX)-mediated oxidation of PUFAs and the biosynthesis of JA. As a result, JA has been identified as a key regulator of plant defense and stress responses [20,21,22,23,24]. However, recent studies have shown that oxylipin groups other than JA are also independent signaling molecules that modulate gene expression, redox homeostasis, hormonal interactions, and developmental processes [25,26,27]. Recently, it has been reported that PXG-dependent pathways mediated by caleosin/PXGs generate distinct oxylipin species, including epoxy and trihydroxy fatty acid derivatives, and contribute to plant responses to abiotic stress [28]. Also, GLVs and non-enzymatically generated PhytoPs are now recognized as biologically active molecules by participating in abiotic stress tolerance in plants [29,30,31,32]. Nevertheless, comprehensive evaluations of the role of these pathways in plant responses to abiotic stress mechanisms remain limited. Another hotly debated area is the crosstalk between oxylipins and other signaling networks. Oxylipin-mediated responses are closely integrated with reactive oxygen species (ROS) signaling, calcium fluxes, mitogen-activated protein kinase (MAPK) cascades, and multiple phytohormone pathways, including abscisic acid (ABA), ethylene, salicylic acid (SA) and brassinosteroids (BRs) [33,34,35,36].

In this review, we provide a comprehensive analysis of the molecular mechanisms of the oxylipin signaling process in plant resistance to abiotic stress. Then, we will discuss the crosstalk of the oxylipin signaling system with other signaling pathways, as well as oxylipin-mediated signaling under major abiotic stresses. Finally, gaps in the current knowledge and the prospects of using oxylipin signaling to ensure crop resilience in global climate change will be discussed.

We identified the literature reviewed in this article through searches of the PubMed, Web of Science, and Scopus databases. Relevant primary research articles and review papers were selected based on their relevance to oxylipin metabolism, signal perception, molecular signaling, crosstalk, and physiological responses to abiotic stress.

## 2. Diversity and Classification of Plant Oxylipins

### 2.1. Biosynthetic Precursors

Plant oxylipins are derivatives of the oxidation of PUFAs formed by lipase-mediated degradation of membrane glycerolipids. They are diverse in structure and function; the dominant precursors are α-linolenic acid (18:3, LnA) and linoleic acid (18:2, LA). Hexadecatrienoic acid (16:3, HTA) is abundant in the chloroplast galactolipid monogalactosyldiacylglycerol (MGDG) of “16:3 plants”, including *Arabidopsis thaliana*, where it serves as a precursor for the biosynthesis of distinct C16-derived oxylipins [17,37]. The availability of these substrates is regulated in a developmental and stress-related manner, reflecting the tight integration of oxylipin metabolism with membrane remodeling [38].

### 2.2. Enzymatically Produced Oxylipins

Enzymatic oxylipin biosynthesis involves several interconnected pathways; among them, the LOX pathway is the best characterized. 9-LOX and 13-LOX catalyze the stereospecific oxygenation of PUFAs at the C-9 and C-13 positions, respectively, generating fatty acid hydroperoxides (FAHPs). The resulting FAHPs act as branch-point intermediates for various downstream enzymes [39]. In the 13-LOX branch, 13-hydroperoxy-α-linolenic acid (13-HPOT) is converted by allene oxide synthase (AOS) to allene oxide, which is subsequently cyclized by allene oxide cyclase (AOC) to form cis-(+)-12-OPDA. This compound subsequently transforms into JA following three cycles of β-oxidation within the peroxisome [40]. Next, JA combines with isoleucine through Jasmonate resistant 1 (JAR1) to form the bioactive jasmonoyl-isoleucine (JA-Ile), which serves as the primary ligand for the CORONATINE INSENSITIVE 1 (COI1) and JASMONATE ZIM-DOMAIN (JAZ) co-receptor complex [41].

9-LOX-mediated oxygenation of PUFAs, particularly LnA, at the C-9 position generates 9-hydroperoxy fatty acids that serve as precursors for diverse oxylipins, including ketols, aldehydes, divinyl ethers, 9-hydroxy-10-oxo-12(Z),15(Z)-octadecadienoic acid (KODA), and other oxygenated lipid derivatives [42,43]. Other LOX-derived hydroperoxides can enter branches leading to ketols, volatile aldehydes and GLVs, divinyl ethers, and other oxygenated fatty acids through enzymes such as hydroperoxide lyase (HPL), divinyl ether synthase (DES), and related hydroperoxide-transforming enzymes [16]. Caleosin/PXGs use FAHPs produced by LOX activity as oxygen donors. PXG-mediated oxygenation results in the formation of hydroxy, epoxy, and hydroxy-epoxy fatty acids [44]. Esterified oxylipins comprise lipid-derived oxygenated fatty acids that remain esterified to membrane lipids rather than occurring as free fatty acids. The best-studied members are arabidopsides, which contain esterified 12-OPDA and dinor-OPDA (dn-OPDA) in chloroplast membranes [45,46]. Additionally, α-dioxygenase (α-DOX) catalyzes the oxygenation of PUFAs at the C-2 position to generate 2-hydroperoxy fatty acids, which are subsequently converted into various α-DOX-derived oxylipins, including 2-hydroxy fatty acids and related oxygenated lipid metabolites [47,48].

### 2.3. ROS-Derived Oxylipins

**Figure 1:**
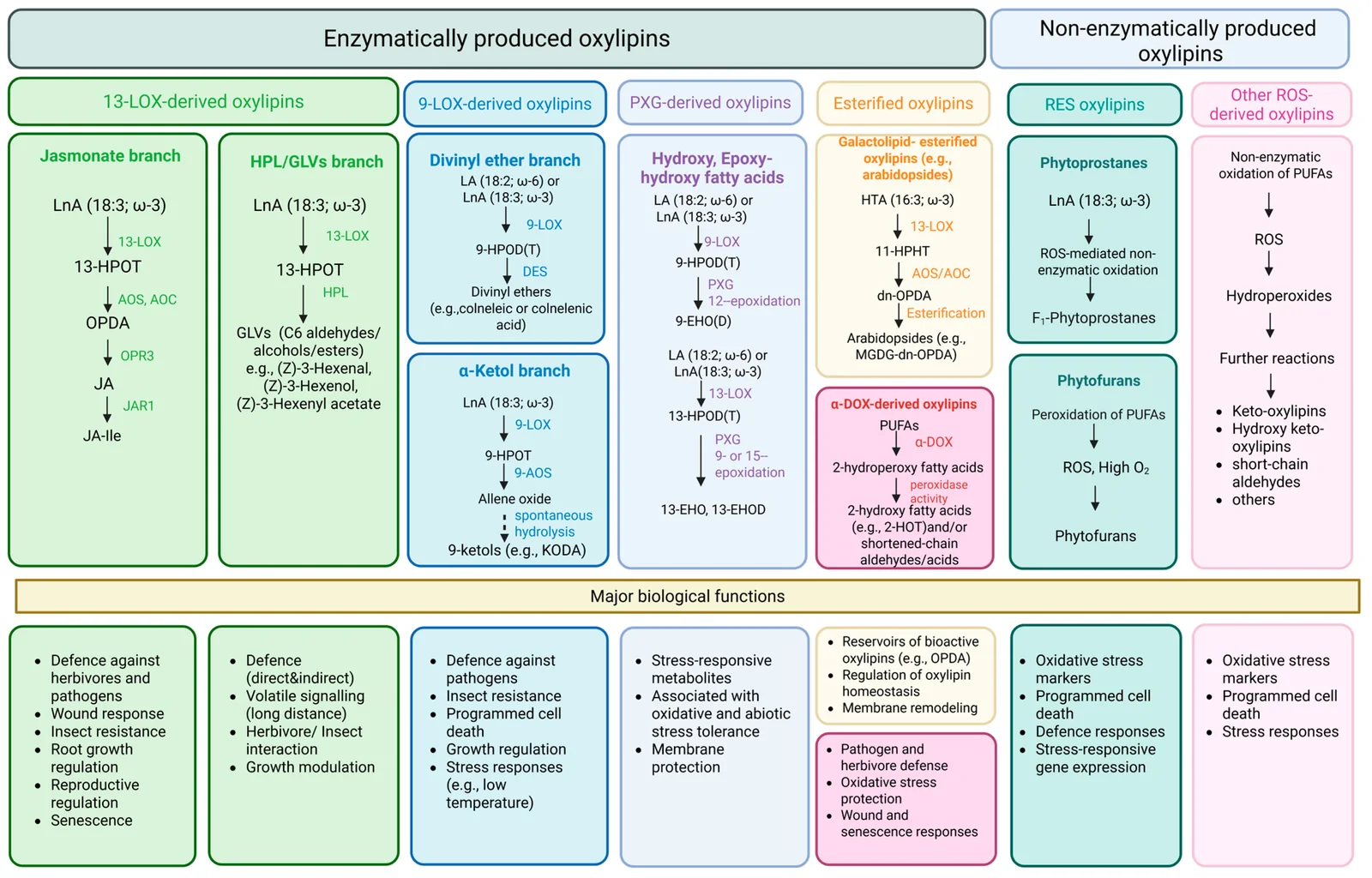
Classification, biosynthetic pathways, and biological functions of major classes of plant oxylipins. Dashed arrows indicate non-enzymatic or spontaneous reactions. Abbreviations. AOC, allene oxide cyclase; AOS, allene oxide synthase; DES, divinyl ether synthase; dn-OPDA, dinor-12-oxophytodienoic acid; EHO, epoxy-hydroxy fatty acid; EHOD, epoxy-hydroxy-octadecadienoic acid; GLVs, green leaf volatiles; HOT, hydroxy-octadecatrienoic acid; HPHT, hydroperoxy-hexadecatrienoic acid; HPL, hydroperoxide lyase; HPOD, hydroperoxyoctadecadienoic acid; HPOT, hydroperoxyoctadecatrienoic acid; HTA, hexadecatrienoic acid; JA, jasmonic acid; JA-Ile, jasmonoyl-isoleucine; KODA, 9-hydroxy-10-oxo-12(Z),15(Z)-octadecadienoic acid; LA, linoleic acid; LnA, α-linolenic acid; LOX, lipoxygenase; MGDG, monogalactosyldiacylglycerol; OPDA, 12-oxophytodienoic acid; OPR3, 12-oxophytodienoate reductase 3; PUFAs, polyunsaturated fatty acids; PXG, peroxygenase; RES, reactive electrophilic oxylipins; ROS, reactive oxygen species; α-DOX, alpha-dioxygenase. Figure was created in BioRender. Eshbekova, G. (2026) https://BioRender.com/moc6e1g.

Non-enzymatically produced oxylipins are generated primarily through free-radical-mediated autoxidation of PUFAs, which is enhanced under oxidative and photooxidative conditions [49]. PhytoPs are plant analogues of mammalian isoprostanes and are formed non-enzymatically through ROS-mediated oxidation and cyclization of LnA in membrane lipids [29]. Phytofurans are structurally related products of non-enzymatic LnA oxidation that arise through an alternative branch of lipid peroxidation, particularly under conditions of elevated oxygen availability [50]. Some PhytoPs, phytofurans, and other reactive carbonyl-containing oxylipins possess electrophilic functional groups, including α,β-unsaturated carbonyl moieties, and act as reactive electrophilic oxylipins (RES). In parallel, non-enzymatic decomposition and rearrangement of PUFA-derived lipid hydroperoxides can generate secondary oxylipins, including keto- and hydroxy-keto fatty acids, as well as short-chain aldehydes and other reactive carbonyl compounds [25] (Figure 1).

### 2.4. Biological Functions of Different Oxylipin Classes

Many excellent review articles have analyzed in detail the biological functions of plant oxylipins, especially jasmonates, including their importance in span development, immunity and environmental acclimation [17,21,22,24,30,49,51,52,53]. However, recent studies have provided deeper insights into the signaling significance of oxylipins in various physiological processes in plants. Jasmonates, including JA and its bioactive conjugate JA-Ile, are multifunctional oxylipin hormones that regulate plant growth, development, defense against herbivores and pathogens, and adaptation to diverse abiotic stresses. In addition to their central roles in wound and immune responses, jasmonates influence processes such as root growth, reproductive development, and senescence [17,24]. OPDA acts partly as a JA precursor but also as an independent signaling molecule; namely, it binds chloroplastic cyclophilin 20-3 (CYP20-3) to regulate sulfur assimilation. Recently, it was reported that its downstream metabolites activate COI1-JAZ-independent gene expression [54,55]. While GLVs primarily function as aerial signals in defense preparation and plant-to-plant communication, some C6 aldehydes additionally serve as reactive electrophiles capable of directly modifying proteins [49,56]. PhytoPs and other RES from the non-enzymatic oxylipin group activate transcriptional programs responsible for detoxification and redox homeostasis [57,58]. Esterified oxylipins, in particular arabidobsides, serve as oxylipin reservoirs present in chloroplast membranes. Following stress or membrane damage, these compounds can produce precursors that contribute to early jasmonate signaling [59]. α-Dioxygenase-derived oxylipins contribute to plant defense and stress adaptation by modulating oxidative stress and promoting appropriate responses to herbivore and pathogen attack [60]. Together, these oxylipin classes form a diverse signaling network in which different molecules perform specific regulatory roles. How these signaling pathways contribute to plant abiotic stress tolerance is discussed further in the following sections.

## 3. Molecular Mechanisms of Oxylipin Signal Perception and Transduction

**Figure 2:**
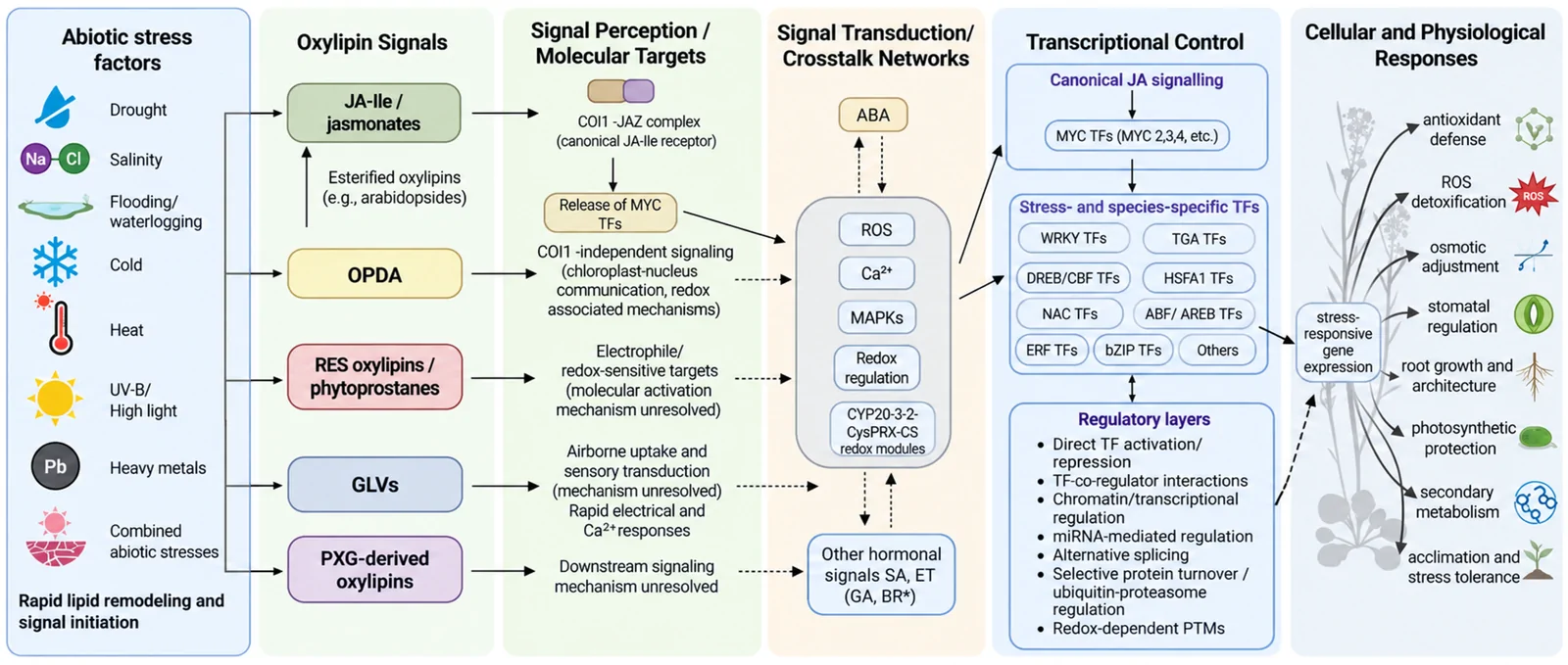
Oxylipin signaling pathways and transcriptional control in plant abiotic stress responses. Solid arrows indicate experimentally established mechanisms, while dashed arrows indicate emerging, indirect, or unresolved relationships. Abbreviations. ABA, abscisic acid; ABF/AREB, ABA-responsive element-binding factor/protein; bZIP, basic leucine zipper (transcription factor); BR, brassinosteroid; Ca^2+^, calcium ion; CBF/DREB, C-repeat binding factor/dehydration-responsive element-binding protein; COI1, coronatine insensitive 1; CYP20-3, cyclophilin 20-3; CysPRX, cysteine-type peroxiredoxin; ERF, ethylene-responsive factor; ET, ethylene; GA, gibberellin; GLVs, green leaf volatiles; HSFA1, heat shock transcription factor A1; JA-Ile, jasmonoyl-isoleucine; JAZ, jasmonate ZIM-domain protein; MAPKs, mitogen-activated protein kinases; MYC, MYC transcription factor (MYC2/3/4, etc.); NAC, NAM/ATAF/CUC transcription factor; OPDA, 12-oxo-phytodienoic acid; PTMs, post-translational modifications; PXG, peroxygenase; RES, reactive electrophile species; ROS, reactive oxygen species; SA, salicylic acid; TFs, transcription factors; TGA, TGACG-binding factor; UV-B, ultraviolet-B radiation; WRKY, WRKY transcription factor. * The crosstalk of GA and BRs with oxylipin signaling remains comparatively less studied. Figure was created in BioRender. Eshbekova, G. (2026) https://BioRender.com/dlrs1xl.

Oxylipins mediate the plant response to abiotic stress by activating multiple signaling pathways that translate changes in lipid metabolism into coordinated cellular responses. Jasmonates are the best-characterized class of oxylipin signaling molecules, but increasing evidence indicates that other oxylipin classes also function as signals, using different or overlapping mechanisms. Depending on their chemical properties, oxylipins can be perceived via receptor-mediated pathways, the electrophilic modification of regulatory proteins or the modulation of cellular redox status. These processes ultimately affect transcriptional reprogramming and stress adaptation. This section summarises our current knowledge of oxylipin perception, signal transduction, transcriptional regulation and the mechanisms ensuring signaling specificity during plant responses to abiotic stress (Figure 2).

### 3.1. Jasmonate Signaling

Among all oxylipin signaling pathways identified in plants, jasmonate signaling is the most comprehensively studied molecular system [33,52,61,62,63,64]. Jasmonate signaling is initiated through ligand-dependent formation of an intracellular COI1–JAZ co-receptor complex. The bioactive jasmonate conjugate JA-Ile is recognized by the F-box protein COI1, a component of the SKP1–Cullin–F-box (SCF) E3 ubiquitin ligase complex. JA-Ile binds within the ligand-binding pocket of COI1, together with an inositol phosphate cofactor, promoting high-affinity association of COI1 with JAZ proteins [41]. COI1 utilizes JA-Ile as a molecular bridge to stabilize the interaction with JAZ proteins. This makes ligand perception dependent on the involvement of both COI1 and the JAZ repressor. JAZ proteins are central negative regulators of jasmonate signaling by contributing to the specificity and amplitude of transcriptional responses through interactions with diverse transcription factors and co-regulators. Under normal conditions, JAZ proteins bind directly to MYC transcription factors via their conserved Jas domain, thereby inhibiting the activation of jasmonate-responsive genes [65,66]. The repression process is further reinforced through the recruitment of co-repressor complexes. Most JAZ proteins interact with the “Novel Interactor of JAZ” (NINJA) adaptor protein via their ZIM domain. In turn, NINJA recruits the TOPLESS (TPL) transcriptional co-repressor via its Ethylene-responsive element-binding factor-associated Amphiphilic Repression (EAR) motif [67]. This indicates that multiple parallel mechanisms are involved in maintaining transcriptional repression prior to stress perception.

Under stress, the increase in JA-Ile levels rapidly shifts the process from repression to activation. The binding of JA-Ile facilitates a stable interaction between COI1 and JAZ proteins. Consequently, the SCF^COI1^ complex ubiquitinates JAZ repressors, which are subsequently degraded via the 26S proteasome [65,66]. The degradation of JAZ proteins releases a number of TFs. Among them, MYC2 acts as a key regulator of jasmonate-dependent gene expression. MYC2 coordinates numerous physiological processes, including responses to abiotic stress, secondary metabolism, root development and the balance between growth and defense [68,69]. Functional redundancy among MYC2, MYC3, and MYC4 contributes to the robustness of jasmonate responses, while differences in their expression patterns and regulatory interactions contribute to context-dependent transcriptional outputs [70]. However, the COI1–JAZ–MYC module forms the core of the jasmonate signaling system, while growing evidence indicates that transcriptional regulation extends far beyond this primary pathway. JAZ proteins interact with various TFs, including members of WRKY, NAC, ERF, MYB, and basic helix–loop–helix (bHLH) families, integrating jasmonate signaling with numerous other pathways involved in development and stress responses [71,72]. Consequently, the transcriptional outcome of JA signaling depends not only on JA-Ile concentration but also on tissue characteristics, the developmental stage and the simultaneous activity of other signaling pathways. Such combinatorial regulation enables plants to generate specific physiological responses while relying on a common perception mechanism.

A key component in the conversion of JA-Ile perception into transcriptional activation is the Mediator complex, specifically its Mediator of RNA polymerase II transcription subunit 25 (MED25). It acts as a molecular bridge between MYC transcription factors and RNA polymerase II. MED25 functions at the interface between JA-Ile perception and transcription by interacting with MYC transcription factors and coordinating their recruitment of the Mediator complex to jasmonate-responsive promoters. JAZ repressors interfere with this transcriptional activation state, whereas JAZ degradation facilitates MYC–MED25-dependent transcription [73,74]. Following the degradation of JAZ proteins, MED25 recruits transcriptional co-activators, including the histone acetyltransferase (HAC1), thereby facilitating chromatin remodeling and the activation of JA-responsive genes [75]. These observations indicate that the jasmonate signaling system operates not merely through the release of TFs but in close association with epigenetic regulation.

Recent studies consistently confirm that the JA signaling system regulates numerous genes involved in responses to biotic and abiotic stress, as well as in processes such as secondary metabolism, lipid metabolism and hormonal crosstalk [10,24,76]. Importantly, transcriptional activation is a dynamic and context-dependent process. The level and duration of JA-responsive gene expression depend on numerous regulatory factors, including the composition of JAZ proteins, functional redundancy within the MYC family, recruitment of the Mediator complex and protein turnover rates. These interconnected mechanisms enable plants to adjust the transcription process in response to the intensity, duration and combination of environmental stresses.

### 3.2. OPDA Signaling

Among plant oxylipins, OPDA occupies a unique position, as it functions both as a direct precursor to jasmonates and as an independent signaling molecule. Although OPDA was initially regarded as an intermediate in JA biosynthesis, a significant number of OPDA-responsive genes are regulated independently of the classical JA-Ile signaling system [77,78]. Unlike JA-Ile, which is recognized by the structurally defined SCF^COI1^–JAZ co-receptor complex, a specific receptor for OPDA has not yet been clearly identified. Existing evidence suggests that OPDA signaling is mediated through multiple pathways rather than a single perception mechanism. Early genetic studies showed that OPDA elicits specific transcriptional responses in *Arabidopsis* mutants defective in JA biosynthesis or perception, indicating that many OPDA-dependent responses occur independently of COI1-mediated signaling [78,79]. Recent studies have refined this model, indicating that OPDA itself may not always be the ultimate signaling molecule, such as tetranor-cis-OPDA (tn-OPDA) and 7-iso-4,5-didehydro-jasmonic acid (4,5-ddh-JA), which activate classic OPDA-responsive marker genes (e.g., ZAT10 and ERF5), independently of the COI1 and MYC TFs [80]. This suggests that OPDA may function not only as a primary signaling ligand but also, at least in part, as a precursor to non-canonical signaling metabolites. In addition, evidence confirms that intracellular compartmentalization strongly influences OPDA signaling. In *Arabidopsis* opr2 opr3 mutants, which are unable to synthesize JA-Ile, exogenously applied OPDA activated numerous stress-responsive genes, whereas the endogenous OPDA pool failed to trigger a distinct transcriptional program under conditions of normal growth or wounding [81]. This discrepancy indicates that the endogenous OPDA pool may be largely sequestered within chloroplast membranes. Subsequently, OPDA is often esterified into galactolipids or rapidly metabolized at that site. Consequently, its availability to cytosolic signal-transducing components is limited. In contrast, exogenously applied OPDA bypasses these spatial constraints and activates signaling pathways that do not operate under normal physiological conditions.

Comparative studies on early land plants confirm that the OPDA signaling system represents an evolutionarily ancient branch of oxylipin biology. In the bryophyte *Physcomitrium patens*, non-targeted metabolomic analysis revealed rapid accumulation of OPDA-related oxylipins following wounding despite the absence of the canonical JA-Ile signaling system found in flowering plants [82]. Similarly, studies in Antarctic moss demonstrated that OPDA coordinates lipid remodeling, flavonoid biosynthesis, antioxidant metabolism and ABA-related responses independently of the COI1–JAZ pathway [83]. These results indicate that the OPDA signaling system was established prior to the emergence of the JA-Ile perception mechanism and likely represents one of the earliest oxylipin-mediated mechanisms coordinating stress adaptation in land plants.

One of the characteristic features of OPDA is its cyclopentenone ring containing an α,β-unsaturated carbonyl group. This electrophilic structure places OPDA within the broader family of RES oxylipins together with PhytoPs and several related metabolites [49]. Cyclopentenone signaling depends largely on the chemical reactivity of electrophilic oxylipins toward nucleophilic residues, particularly protein cysteine thiols. Comparative studies have shown that OPDA and PhytoPs activate overlapping transcriptional programs independently of both COI1 and MYC2 [57,84]. The electrophilic cyclopentenone moiety determines signaling activity. Consistent with this view, activation of the GST6 promoter occurs in response to electrophilic cyclopentenones but not to chemically similar non-electrophilic cyclopentanones, including JA [57]. These findings indicate that electrophilic reactivity itself constitutes an important mechanism of signal initiation.

One of the best-characterized molecular targets of OPDA is CYP20-3, a chloroplast-localized protein involved in redox regulation. Biochemical studies have shown that OPDA binds directly to CYP20-3, facilitating the formation of the cysteine synthase complex. Subsequently, under stress conditions, this interaction enhances sulfur assimilation and glutathione biosynthesis [55]. This interaction represents one of the clearest mechanistic links between OPDA accumulation and downstream metabolic responses. However, recent data indicate that activation of the CYP20-3 pathway primarily occurs following exposure to exogenous OPDA. This suggests that the pathway may function during detoxification or under conditions of high OPDA levels, rather than serving as a primary endogenous signaling mechanism [81]. Recent studies have further refined this signaling model, demonstrating that reduced glutathione (GSH) acts as a metabolic signal linking OPDA perception to chloroplast metabolism. The interaction between OPDA and CYP20-3 stimulates sulfur assimilation and cysteine biosynthesis, ultimately increasing GSH accumulation. GSH coordinates photosynthetic activity with the activation of defense mechanisms by balancing carbon assimilation and stress-responsive metabolism [85]. These results confirm that OPDA signals link chloroplast redox regulation, primary metabolism and defense responses through metabolite-mediated signaling.

Downstream of cyclopentenone perception, proteins belonging to the TGA subfamily of basic leucine zipper (bZIP) TFs emerged as key regulators of the electrophilic oxylipin responses. TGA2, TGA5 and TGA6 factors are essential for the activation of numerous genes involved in detoxification and the response to oxidative stress triggered by OPDA and PhytoPs [84]. These TFs regulate genes involved in glutathione metabolism, xenobiotic detoxification and antioxidant defense. Although TGA2 contains a reactive cysteine residue susceptible to modification by cyclopentenones, this residue is not critical for the activation of detoxification genes in response to OPDA [57]. Consequently, direct electrophilic modification of TGA proteins is unlikely to represent the primary mechanism of transcriptional activation. Instead, current evidence suggests that additional upstream signaling components relay electrophilic signals to the TGA transcriptional machinery. Supporting this hypothesis, a recently identified CW-type zinc finger protein has been proposed as a novel regulator of RES-mediated signaling and response to abiotic stress in *Arabidopsis*, despite its precise position within the signaling cascade remaining to be determined [58].

Generally, OPDA contributes positively to stress adaptation; however, its effects appear to be context-dependent. In rice, mutants with limited OPDA accumulation due to a deficiency in the AOC enzyme exhibited enhanced salt tolerance. This response was accompanied by an increased capacity to scavenge ROS and heightened antioxidant enzyme activity [86]. These results suggest that, under certain physiological conditions, the attenuation of OPDA signaling can mitigate oxidative damage through the compensatory activation of antioxidant pathways. Current evidence indicates that OPDA signals are integrated into broader redox regulatory networks, that its physiological impact depends more on the species, type of stress and metabolic conditions than on playing a universally positive role in stress tolerance.

### 3.3. Post-Transcriptional and Post-Translational Regulation of Oxylipin Signaling

Oxylipins also induce post-transcriptional regulatory mechanisms, amplifying the transcriptional pathways induced by oxylipins. These mechanisms are not limited to the activation of TFs, but also involve miRNA-mediated gene silencing, alternative splicing and selective protein degradation. Together, they provide additional regulatory levels to allow plants to fine-tune oxylipin signaling to stress intensity, duration and developmental context. While most mechanistic understanding has been derived from studies of jasmonate signaling, recent research indicates that these regulatory mechanisms also extend to other oxylipin-mediated stress responses.

MicroRNAs have been identified as important regulators that link jasmonate signals to networks of plant development and environmental responses. Many miRNAs have also been identified as participants in jasmonate-mediated stress response pathways, suggesting extensive crosstalk between small RNA pathways and hormone signaling systems [87]. Most miRNAs target TFs and signaling proteins downstream of JA-Ile perception. One of the best characterised examples is miR319 that targets members of the Teosinte branched1/Cycloidea/Pcf (TCP) TF family. It modulates the expression of jasmonate-responsive genes and stress adaptation [88]. Functional studies have shown the significance of the miR319–TCP regulatory module in providing abiotic stress tolerance in various plant species. Constitutive expression of OsmiR319a in *Agrostis stolonifera* creeping bentgrass improved drought and salinity tolerance through downregulation of a series of TCP TFs and modulation of the activity of stress-responsive downstream genes, such as a NAC-family TF [89]. In sweet potato, disruption of the miR319–TCP module reduced drought tolerance and altered stomatal development and lignin biosynthesis. These findings demonstrate the role of this module in coordinating developmental plasticity with stress adaptation processes [90]. Although downstream targets vary across species, the miR319 TCP regulatory network is highly conserved in integrating growth and abiotic stress response processes. Recently, a more direct mode of regulation within the jasmonate signaling pathway was identified in wheat. The miR1119–MYC2 module responds strongly to drought stress and exhibits contrasting expression patterns between drought-tolerant and drought-sensitive cultivars. Changes in miR1119 expression are associated with ABA accumulation, photosynthetic efficiency, antioxidant capacity and plant water status [91]. This indicates that MYC2 itself may be regulated at the transcript level via miRNA-mediated silencing. Consequently, miRNA-mediated regulation complements the classical COI1–JAZ–MYC signaling module. GLVs perception has also been linked to a transcriptional output; (E)-2-hexenal induces WRKY40 and WRKY6 expression as downstream regulators of hexenal-responsive genes in Arabidopsis [92].

Alternative splicing provides another mechanism for diversifying the effects of oxylipin signaling without altering the genomic sequence. Environmental stress often affects splice site selection, leading to the generation of transcript variants with differences in protein structure, stability or regulatory activity [93,94]. In the jasmonate pathway, alternative splicing has been most extensively studied in the JAZ family of repressor proteins. In most plant JAZ genes, the Jas motif is interrupted by a highly conserved intron. Retention of this intron results in truncated splice variants lacking the conserved C-terminal X_5_PY sequence required for effective interaction with COI1 [95]. These truncated proteins are still able to bind MYC TFs, but they have a low affinity for the COI1 receptor and thus evade efficient JA-Ile-triggered, SCF^COI1^-mediated degradation. Thus, they function as relatively stable repressors of jasmonate signaling. The JAZ10.4 isoform, generated by alternative splicing, does not possess a full Jas motif, but is able to interact with MYC2, MYC3, MYC4 and the adapter protein NINJA [96]. Since this splicing variant evades COI1-mediated degradation while retaining its transcriptional repression activity, it establishes a negative feedback mechanism that limits the prolonged activation of jasmonate-responsive genes. Such a regulatory mechanism may be particularly important under conditions of sustained abiotic stress; otherwise, continuous hormonal signaling could adversely affect plant growth and resource allocation.

Selective protein degradation is one of the most rapid mechanisms for regulating oxylipin signaling. The significance of regulated proteolysis became evident when it was discovered that, following the perception of JA-Ile, JAZ repressor proteins are rapidly degraded via the SCF^COI1^ ubiquitin-ligase complex. This releases MYC TFs and initiates transcriptional reprogramming [65,66]. However, recent studies indicate that protein turnover within the oxylipin signaling network extends well beyond this classical pathway. Additional ubiquitin ligases selectively regulate specific components of the jasmonate signaling cascade. In tomatoes, the U-box E3 ubiquitin ligase PUB22 provides an additional level of post-translational control by increasing the rate of JAZ4 protein degradation by the 26S proteasome, in a manner independent of the SCF^COI1^ complex. MYC2 directly activates PUB22 transcription, establishing a positive feedback loop in which JA signaling promotes specific degradation of JAZ4, thus further promoting transcriptional activation [97]. This finding suggests that different members of the JAZ family could be targeted by different degradation pathways.

The stability of transcription activators is also tightly regulated. The CUL3–BPM ubiquitin-ligase complex targets MYC2, MYC3 and MYC4 proteins for proteasomal degradation, thereby limiting the over-amplification of jasmonate signaling following stress exposure. This negative regulatory mechanism provides an additional layer of control over jasmonate-responsive transcription by limiting the abundance of MYC transcription factors and preventing prolonged pathway activation [98]. In contrast to the canonical JA pathway, the post-transcriptional or post-translational regulation of non-JA-related oxylipin signaling relies on the electrophilic properties of reactive oxylipins. OPDA covalently modifies cysteine residues on numerous redox-sensitive proteins through OPDAylation, establishing electrophilic modification as a distinct regulatory mechanism in RES signaling [99]. Since JA-Ile lacks the α,β-unsaturated carbonyl group, this type of thiol modification appears to be specific to electrophilic oxylipins. In addition, RES oxylipins, including OPDA and PhytoPs, activate HSFA1-dependent heat stress transcription independently of COI1 and JA-Ile, resulting in the rapid induction of heat shock genes such as HSP101. This response is not observed in the hsfA1 abde quadruple mutant, indicating that RES oxylipins activate a transcriptional program distinct from the classical jasmonate pathway [100].

Overall, oxylipin signaling is regulated through interconnected transcriptional, post-transcriptional, and post-translational mechanisms. Nevertheless, the molecular basis of signal perception and the downstream signaling events remain incompletely resolved for other emerging oxylipin signaling pathways. Existing data indicate that these oxylipins often utilize established stress-signaling networks. How such interactions are integrated with other stress-responsive pathways is therefore an important aspect of oxylipin-mediated stress adaptation and will be discussed further in the following sections.

## 4. Crosstalk Between Oxylipins and Cellular Signaling Networks

Oxylipins operate within a highly interconnected signaling network that integrates redox signals, calcium fluxes, protein phosphorylation cascades and multiple phytohormone pathways. Upon the perception of abiotic stress, these signaling systems are activated almost simultaneously and communicate via common molecular regulators, enabling plants to rapidly coordinate stress perception. The following section focuses on molecular mechanisms underlying signaling integration of major oxylipin classes, as well as the recent advances in jasmonate signaling (Table 1).

### 4.1. ROS–Oxylipin Crosstalk

ROS are among the first signaling molecules produced in response to environmental stress. They play a pivotal role in starting oxylipin-mediated signaling processes. Environmental stresses like drought, salinity, extreme temperatures, flooding, and heavy metals quickly disrupt the balance of cellular redox, leading to the buildup of superoxide (O_2_^•−^), hydrogen peroxide (H_2_O_2_), singlet oxygen (^1^O_2_), and hydroxyl radicals (•OH) [101,102]. While high levels of ROS can damage cellular structures, their temporary buildup serves as an important signal for activating defense responses.

The first step in this process involves the lipid peroxidation of PUFAs in chloroplasts and plasma membranes. This reaction provides substrates for LOX-dependent and ROS-mediated oxylipin formation. As a result, ROS can influence both the level of oxidative stress and the types of oxylipin signals generated during the stress adaptation period.

The RES oxylipins are an important link between oxidative stress and the subsequent signal transduction processes. They act as signaling molecules that convert the lipid peroxidation initiated by ROS into changes in protein activity and gene expression [103]. The biological activity of RES signaling molecules does not depend on receptor binding; it relies on their ability to participate in chemical reactions. Their electrophilic α,β-unsaturated carbonyl group allows for the specific modification of reactive cysteine residues in regulatory proteins.

This changes the activity, stability, or interactions of the proteins. This signaling mechanism enables lipid oxidation products created by ROS to be transformed into immediate redox-dependent cellular responses [104,105]. During abiotic stress, ROS production mainly occurs through Respiratory Burst Oxidase Homologs (RBOH), specifically RBOHD and RBOHF. These proteins catalyze the formation of apoplastic superoxide and trigger local and systemic ROS waves [106,107]. ROS generated in chloroplasts further increases the oxidation of membrane lipids. This, in turn, encourages both enzymatic oxylipin biosynthesis and the non-enzymatic formation of PhytoPs and phytofurans. Therefore, the strength and length of ROS buildup affect the relative amounts of different oxylipin classes. This suggests that the cellular redox state is not just a trigger for lipid oxidation but also an important factor that shapes the oxylipin profile.

The interaction between ROS and oxylipins is bidirectional; while ROS stimulate oxylipin production, several classes of oxylipins subsequently regulate ROS generation. Jasmonate signaling connects to the activation of ROS production, which mainly relies on RBOH. JA creates a feedback loop that boosts oxidative signaling, which might further improve oxylipin biosynthesis [107,108]. Genetic evidence shows that RBOHD-dependent ROS production and jasmonate signaling work together during various stress responses. These include managing stomatal regulation and signaling related to wounds. In Arabidopsis, both RBOHD-mediated ROS production and COI1-dependent jasmonate signaling are needed for quick stomatal responses. RBOHD also plays a role in accumulating systemic JA and signaling wounds over long distances [109,110].

OPDA mainly regulates ROS homeostasis through redox mechanisms tied to chloroplasts. When OPDA binds to CYP20-3, it boosts sulfur assimilation and cysteine production. This leads to more glutathione and better redox buffering [55]. GSH then serves as a metabolic signal that coordinates photosynthesis with defense responses. This illustrates how OPDA connects primary metabolism with stress signals [85]. Recent studies have identified a new layer of regulation; OPDA can chemically modify thioredoxins and glutaredoxins through OPDAylation. This reversible, redox-dependent change can temporarily suppress antioxidant activity in chloroplasts and extend the duration of ROS signaling [99,111]. The physiological importance of OPDAylation during abiotic stress is still under examination, but these findings suggest that OPDA manages redox homeostasis through both metabolic and post-translational mechanisms.

The integration between ROS and oxylipins also involves transcriptional regulation. Changes in the cellular redox state affect the activity of several TFs through reversible oxidation of cysteine residues and shifts in glutathione levels and TFs including TGA, WRKY, NAC, bZIP and DREB are broadly redox-responsive [112,113]. Among these, TGA factors have been directly linked to oxylipin signaling; PhytoPs and OPDA activate TGA-dependent detoxification gene expression independently of the classical COI1-mediated pathway [84].

Antioxidant defense systems play a central role in maintaining ROS homeostasis during oxylipin-associated stress responses. Oxylipin accumulation is frequently associated with increased activity of superoxide dismutase (SOD), catalase (CAT), ascorbate peroxidase (APX), glutathione peroxidases (GPX), glutathione S-transferases (GST), and enzymes of the ascorbate–glutathione cycle [114,115,116], and transgenic manipulation of oxylipin biosynthesis has been shown to directly alter antioxidant enzyme activity, supporting a causal contribution to this relationship [117].

Although PXG-derived oxylipins remain considerably less characterized, recent evidence suggests that PXGs are directly involved in the regulation of cellular redox homeostasis under abiotic stress. OsPXG9 acts as a negative regulator of ROS accumulation and confers oxidative stress tolerance in rice. OsPXG9 loss caused over-accumulation of ROS and increased oxidative damage under abiotic stress, while increased OsPXG9 activity increased the cellular resistance due to the maintenance of ROS homeostasis [27].

### 4.2. Calcium–Oxylipin Crosstalk

Ca^2+^ is another early signaling component that is closely linked to oxylipin signaling. Within seconds of sensing stress, short bursts of cytosolic Ca^2+^ generate specific calcium signatures that encode information on the strength and type of environmental signals [118,119]. These patterns result from the coordinated action of plasma membrane and intracellular calcium channels including glutamate receptor-like channels (GLRs), cyclic nucleotide-gated channels (CNGCs) and osmosensitive calcium-permeable channels (OSCAs). Together, they translate membrane disturbance to intracellular signaling events [120,121].

One of the first effects of Ca^2+^ influx is the activation of membrane lipid remodeling. Calcium-dependent phospholipases release PUFAs from membrane phospholipids, increasing the availability of substrates for oxylipin production [122]. Blocking Ca^2+^ influx significantly reduces wound-induced jasmonate buildup, showing that calcium signaling is important not only for lipid mobilization but also for activating oxylipin biosynthetic genes [121]. Thus, calcium works before oxylipin signaling by linking stress perception with membrane remodeling and oxylipin production.

This relationship also works both ways; several oxylipins trigger specific calcium responses that enhance downstream signaling. Among the different classes of oxylipins, GLVs provide some of the clearest examples of calcium-linked signaling, triggering rapid Ca^2+^ transients that support their proposed role as early stress signals [32]. Exposure to (Z)-3-hexenal and (E)-2-hexenal quickly raises cytosolic Ca^2+^ levels and activates calcium-dependent signaling pathways in Arabidopsis. In contrast, related C_6_ compounds show much weaker activity [123]. These findings indicate that GLV perception has some structural specificity, even without an identified receptor.

OPDA and jasmonates also affect calcium-dependent signaling, but these responses are mainly routed through downstream signaling networks [55]. The information encoded within calcium signatures is interpreted by calcium sensor proteins, including calmodulins (CaMs), calmodulin-like proteins (CMLs), calcium-dependent protein kinases (CDPKs/CPKs), and calcineurin B-like protein (CBL)-interacting protein kinase (CIPK) complexes [119]. These calcium decoders connect transient Ca^2+^ signals with phosphorylation cascades and transcriptional regulation. Although relatively few studies have examined their direct interaction with oxylipin signaling, current evidence suggests that CDPKs and CBL–CIPK complexes regulate several TFs that also function downstream of oxylipins, including MYC, WRKY, NAC, and DREB proteins. This leads to the suggestion that calcium signaling may contribute to the quantitative regulation of oxylipin-responsive gene expression [69,124,125].

At the cellular level, calcium signaling also provides an important link between local and systemic responses. Calcium waves can propagate rapidly through vascular tissues following local stimulation and contribute to long-distance signaling, where they interact with other systemic signals [121]. In guard cells, Ca^2+^ signaling is also functionally integrated with jasmonate and ROS pathways to regulate wound-induced stomatal closure, illustrating that these signals can act through interconnected mechanisms [126].

### 4.3. MAPK Cascades in Oxylipin Signaling

MAPK cascades are important signaling modules that connect the oxylipin signaling system to transcriptional responses. MAPKs act as signal integrators and amplifiers, conveying information from various stress-related pathways, including ROS, Ca^2+^ signals, receptor-like kinases and phytohormones, to regulate TF activity and expression of stress-responsive genes [127,128]. The canonical MAPK cascade consists of three sequentially activated kinases: MAPK kinase kinases (MAPKKKs), MAPK kinases (MKKs) and MAPKs. Upon stress perception, phosphorylation events are relayed through this cascade activating downstream regulatory proteins. Among the best-studied representatives, MPK3, MPK4, and MPK6 are consistently implicated in the oxylipin signaling system and abiotic stress responses [129,130]. Although these kinases participate in several signaling pathways, recent studies indicate that they also contribute to oxylipin-mediated signaling processes.

Oxylipins influence MAPK activity through several mechanisms. Jasmonates rapidly induce phosphorylation of MPK3 and MPK6, resulting in activation of TFs involved in stress-responsive gene expression [131]. Recent analyses indicate that GLVs activate signaling pathways associated with the perception of damage-associated molecular patterns (DAMPs). Exposure of *Solanum peruvianum* suspension-cultured cells to (Z)-3-hexen-1-ol or (Z)-3-hexenyl acetate rapidly triggered changes in the phosphorylation status of pattern recognition receptors, a receptor-like cytoplasmic kinase, MAPK cascade components, calcium signaling proteins, and transcriptional regulators within five minutes of treatment [132].

GLV-mediated activation of MAPK signaling has also been demonstrated in grasses, where exposure to volatiles released from damaged tissue rapidly induces the phosphorylation of at least two MAPK isoforms within minutes of perception. The kinetics of this response differ from those observed following direct mechanical wounding in the same species: GLV-induced MAPK activation is transient and attenuates rapidly despite continued volatile exposure, whereas wound-induced MAPK activation is more sustained. This suggests that GLVs function primarily as priming signals that sensitize receiver tissue to subsequent stress stimuli [133]. This capacity of GLV-mediated MAPK activation appears conserved across evolutionarily diverse grass species [134], even though the upstream receptor and kinase components responsible for GLV perception and MAPK cascade recruitment have not been identified. GLV signaling can modify the sensitivity of jasmonate-associated MAPK responses. Although methyl jasmonate (MeJA) alone does not induce MAPK phosphorylation, prior jasmonate exposure enhances the MAPK response to subsequent wounding. These findings suggest that the two oxylipin-related signals interact by modifying MAPK pathway sensitivity [133].

PhytoPs also participate in MAPK-related signaling processes. PhytoPs isomers including PPA1-I/II, deoxyPPJ-I/II and PPB1-II enhanced the activation of MAPKs upon their exogenous application in tomato cell culture and this response varied among the isomers; for example, PPB1-I induced little or no activation [30,135]. This finding indicated that MAPK activation by PhytoPs depends on the chemistry and stereochemistry of their structure. In addition, there is evidence showing that RES-oxylipins modulate protein phosphorylation networks. Electrophilic oxylipins induce rapid changes in the expression of stress-responsive genes, which are correlated with the activation of kinase-dependent signaling pathways. It is hypothesized that RES-oxylipins can act directly with intracellular signal transduction proteins by forming covalent bonds as electrophiles, affecting downstream phosphorylation pathways [103].

MAPKs also participate in feedback regulation of jasmonate signaling. JA itself activates the MKK3–MPK6 cascade, which in turn negatively regulates *MYC2/JIN1* expression and JA-induced root growth inhibition, establishing a negative feedback loop that limits MYC2-dependent transcriptional output [131]. In tobacco, wound-induced activation of the MAPK homolog WIPK correlates with increased JA accumulation, suggesting positive contribution to wound-induced jasmonate biosynthesis [136]. One of the primary functions of MAPKs is the phosphorylation of TFs that regulate oxylipin-responsive genes. WRKY TFs are the best-characterized substrates for MAPKs and play a crucial role in coordinating the expression of stress-related genes [137]. Experimental evidence confirms that MAPKs regulate the activity of MYC2, ERF, NAC, DREB and bZIP TFs either directly or indirectly through intermediate regulatory proteins and signaling pathways [69,128]. MAPKs function not merely as independent controllers of specific signaling pathways, but as integrators and amplifiers of signals from diverse sources prior to influencing common transcriptional regulators. Because MAPKs are activated by ROS, Ca^2+^, and various phytohormones, they constitute a critical convergence point within the oxylipin signaling network. This central position enables MAPKs to integrate diverse environmental signals into coordinated transcriptional responses while maintaining the plasticity of the signaling process.

### 4.4. Oxylipin Crosstalk with Phytohormone Signaling Networks

Multiple hormonal pathways share signal transduction components, TFs, protein kinases and regulatory proteins through an interconnected regulatory network of oxylipin signaling. These interactions are mainly at the downstream levels and are mediated by common transcriptional regulators and post-transcriptional mechanisms. Thus, plants are able to integrate developmental and environmental cues into coordinated gene expression programs while maintaining signal specificity (Table 1).

ABA is one of the most functionally connected plant hormones to jasmonate signaling under abiotic stresses. While ABA and oxylipins are sensed by different receptor systems, they share downstream signaling pathways converging on several common regulatory components. One of the most studied links is via the MYC2 TF, which regulates genes responsive to both ABA and JA and is capable of integrating signals from the two pathways [62,138]. Recent studies have shown that this crosstalk is mediated through common regulatory proteins. Pepper JAZ protein CaJAZ1-03 physically interacts with RING-type E3 ubiquitin ligase CaASRF1 to form a regulatory module. This module governs ABA signaling and drought response reactions by controlling the stability of the JAZ protein and establishes a direct molecular link between jasmonate and ABA signaling pathways [139]. In melons, drought-induced accumulation of ABA, H_2_O_2_ and JA levels leads to the concerted activation of CmCAD genes involved in lignin biosynthesis as well [140]. Besides jasmonates, evidence is also available for the involvement of caleosin/PXG proteins in ABA-associated signaling processes. Several caleosins in Arabidopsis are regulated by ABA at the transcriptional level. Genetic analyses show that they influence ABA-responsive gene expression. For instance, the expression of the ABA-responsive transcription factors ABF3 and ABF4 was up-regulated in plants with disrupted AtCLO4 function, but down-regulated in plants overexpressing AtCLO4. This suggests that AtCLO4 negatively regulates ABA signaling [141]. Similarly, studies of AtCLO3 (RD20) and ABA-related mutants suggest that PXG-associated lipid metabolism is also involved in ABA-dependent stress responses [142,143].

Jasmonate signaling is coordinated with ethylene pathways mainly by interactions between JAZ repressors, MYC2, EIN3/EIL1 and ERFs. Together, they regulate common sets of stress-responsive genes. Under normal conditions, JAZ proteins repress MYC TFs and regulate EIN3/EIL1 activity, thereby limiting transcriptional responses. Upon the accumulation of JA-Ile, JAZ proteins are degraded via the SCF^COI1^ complex. Consequently, MYC2 is released, enabling the coordinated regulation of ERF-dependent transcription [144,145]. MED25 further enhances this integration by coordinating the crosstalk between MYC2- and ERF-dependent transcriptional networks [75]. Recent experimental studies have demonstrated that this signaling module is also involved in adaptation to abiotic stress. ERF.D2 acts as a negative regulator of drought tolerance in tomato by integrating ABA and jasmonate signaling [146]. The evidence provided supports that ethylene-oxylipin crosstalk is embedded within a larger hormone signaling network mediating the integration of numerous environmental signals into adaptive and coordinated transcription responses [10,145].

The balance between jasmonate and SA signaling contributes to the coordination of plant responses to environmental stress. An inverse relationship between JA and SA accumulation has been observed during the early wound response in rice, suggesting negative crosstalk between these two hormonal pathways during stress signaling [147]. Metabolomic analyses in *Clematis terniflora* further revealed that UV-B radiation and darkness altered the relative accumulation of JA and SA, suggesting that modulation of their hormonal balance is associated with metabolic reprogramming during stress adaptation [148]. Furthermore, jasmonates and SA undergo coordinated metabolic and signaling changes during oxidative stress and interact through redox-regulated signaling networks [149].

The molecular interactions between BRs and other groups of oxylipins, except jasmonates, as well as their crosstalk with auxins, have not been well characterized. In brassinosteroid signaling, the signal is initiated through receptor kinase Brassinosteroid insensitive1 (BRI1) and its co-receptor BRI1-associated receptor kinase 1 (BAK1), which then activate phosphorylation cascade, resulting in inhibition of Brassinosteroid-insensitive 2 (BIN2) kinase and accumulation of the TFs BES1 and BZR1 in nucleus [150,151]. MYC2 has been shown to physically interact with BZR1, disrupting its association with Phytochrome-interacting factors (PIF) TFs and repressing BR-responsive gene expression during apical hook development in Arabidopsis [152]. This establishes JA–BR crosstalk in this developmental context as predominantly antagonistic, with MYC2 acting as a direct repressor of BZR1 signaling output. This antagonistic relationship is consistent with broader characterizations of JA–BR crosstalk, which is generally understood to oppose brassinosteroid-driven growth processes as part of resource allocation between growth and defense in plants [153]. The interaction between auxin and jasmonate signaling systems is also based on a similar organizational principle. The MYC2 TF functions as a central controlling center by coordinating jasmonate-inducible transcription with auxin-regulated morphogenesis. MYC2 negatively affects the expression of root meristem-sustaining PLETHORA(PLT)genes, connecting the jasmonate pathway to auxin-dependent root development [154]. The auxin signaling induces the JAZ1 expression through an Auxin response factor (ARF)-dependent mechanism which makes a direct regulation between the two signal pathways [155]. Jasmonates also regulate the accumulation and localization of PIN-FORMED(PIN) auxin transporters. In turn, this connects the stress signaling to Auxin transport and developmental plasticity [156].

Interaction between the oxylipin and GA signaling pathways primarily occurs during regulation of transcription to growth and stress responses. Present findings suggest that the jasmonate signal pathway intersects with the GA pathway through the mutual regulation of JAZ repressors and DELLA proteins, key regulators of GA signaling [157,158]. DELLA proteins physically interact with JAZ repressors, reducing their inhibitory effect on MYC2 and thereby enhancing the expression of jasmonate-responsive genes. Conversely, the accumulation of JAZ proteins can alleviate DELLA-mediated growth inhibition. This demonstrates a mechanism of mutual regulation that balances the activation of defense mechanisms with growth processes. Although many mechanistic studies have primarily focused on the regulation of developmental processes, growing evidence indicates that the JAZ–DELLA–MYC2 module also contributes to adaptation to abiotic stress by coordinating transcriptional programs associated with the regulation of cellular defense and growth [62,69]. Evidence regarding the crosstalk between oxylipin metabolism and gibberellin signaling has also been provided by studies on the caleosin/PXG protein Responsive to desiccation20 (RD20), which is involved in the metabolism of FAHs [143]. Disruption of RD20 activity altered GA-dependent flowering time, increased sensitivity to ABA, and reduced tolerance to oxidative stress. This indicates that PXG-associated lipid peroxide metabolism contributes to hormone-regulated development and stress responses. However, the precise signaling function of the oxylipins produced by PXG has not yet been fully established; these results suggest that caleosin/PXG proteins may serve as an additional point of integration between oxylipin metabolism and gibberellin signaling.

Although oxylipins are produced via diverse biosynthetic pathways and their perception mechanisms vary, their signal transduction pathways converge at a relatively small number of regulatory hubs. Within the signaling network, proteins such as MYC2, JAZ repressors, WRKY and TGA transcription factors, MAPKs, calcium-dependent protein kinases, and redox-sensitive regulators repeatedly emerge as central integrating elements that coordinate information derived from ROS, calcium, MAPKs, and numerous phytohormones. These pathways function not as independent signaling modules but as a dynamic regulatory network, with individual signaling components participating in multiple molecular interactions.

## 5. Roles of Oxylipins in Specific Abiotic Stress Responses

Oxylipin-mediated signaling processes rely on conserved molecular mechanisms, and the functions of specific oxylipin classes vary to some extent under different abiotic stress conditions. Environmental stresses, such as drought, salinity, waterlogging, heat, cold, heavy metals, and UV-B radiation, lead to the differential accumulation of oxylipin classes. As mentioned in the previous section, oxylipins interact extensively with signaling networks involving ROS, calcium, MAPK cascades and phytohormones. However, their contribution to plant adaptation processes depends on the specific type of abiotic stress and the resulting plant responses. The following section summarizes the key mechanisms of oxylipin-mediated responses to individual and combined abiotic stresses and Table 2 presents significant experimentally validated studies.

### 5.1. Water-Related Stresses

#### 5.1.1. Drought

Water deficit leads to rapid remodeling of membrane lipids. As a result, this process initiates the biosynthesis of various oxylipins, which serve as key regulators in drought adaptation (Table 2). Activation of the LOX pathway is considered one of the earliest biochemical responses to water deficit. The upregulation of oxylipin biosynthesis occurs concurrently with extensive metabolic and transcriptional processes within the cell, indicating that lipid-derived signals are integral to this adaptive network [159]. Research in this area demonstrates that drought-induced jasmonate accumulation is tightly regulated through the coordinated activity of LOX, AOS, AOC and OPR enzymes [160,161]. Downstream signal transduction is mediated by the COI1–JAZ–bHLH regulatory module. The degradation of OsJAZ repressors releases the OsbHLH148 protein, thereby activating OsDREB1A, a drought-responsive gene in rice [162]. OsJAZ1 acts as a negative regulator of drought tolerance by modulating the interplay between JA and ABA [163]. Similarly, OsWRKY76 positively regulates drought adaptation during JA-dependent signaling by activating drought-responsive transcription in cooperation with OsbHLH148 [164]. Observations of mutants with impaired JA synthesis clearly show the crosstalk between jasmonate and ABA pathways, as defects in JA biosynthesis affect ABA accumulation and attenuate response reactions to drying soil [165].

In recent decades, it has been determined that other groups of oxylipins also perform specific functions during adaptation to drought. For instance, OPDA promotes ABA-dependent stomatal closure and influences root development without requiring conversion into jasmonates [166,167]. Meanwhile, oxylipins generated via the 13-LOX pathway participate in root-shoot communication, facilitating the transmission of drought-related signals [168]. Additionally, the PXG-associated protein RD20 (AtCLO3) regulates transpiration and water loss through oxylipins produced by PXG [169]. Another interesting recent study found that the exogenous application of the GLV (Z)-3-hexenyl acetate also enhances drought tolerance [170].

**Table 2:** Experimentally validated roles of oxylipins in plant abiotic stress responses.

Abiotic Stress	Oxylipin	Species	Key Molecular Component(s) (Gene, etc.)	Experimentally Validated Role	Ref.
Drought	JA	*Cicer arietinum*	LOX, AOS, AOC, OPR; endogenous JA	Early drought induces JA biosynthesis and accumulation in roots	[160]
	JA	*Cucumis melo*	CmLOX10	Positively regulates JA accumulation, promotes stomatal closure and enhances drought tolerance	[161]
	JA	*Oryza sativa*	OsbHLH148, OsJAZ proteins	Upon JA-induced degradation of OsJAZ repressors, OsbHLH148 activates drought-responsive genes, including OsDREB1A, thereby enhancing drought tolerance.	[162]
	JA	*Oryza sativa*	OsJAZ1	Negative regulator of drought tolerance via JA–ABA signaling	[163]
	JA	*Oryza sativa*	OsWRKY76, OsbHLH148	OsWRKY76 enhances drought tolerance through JA signaling	[164]
	JA/JA-Ile	*Arabidopsis thaliana*	JA-deficient mutants (aos, opr3, jar1-1)	Reduced endogenous jasmonate accumulation altered ABA accumulation and drought responses using JA-deficient mutants	[165]
	OPDA	*Arabidopsis thaliana*	OPDA, ABA	12-OPDA promotes ABA-dependent stomatal closure during drought	[166]
	OPDA	*Avena sativa*	OPDA	Modulates root growth and contributes to drought tolerance independently of JA	[167]
	JA	*Arabidopsis thaliana*	Endogenous JA and ABA	Early hormonal reprogramming contributes to drought acclimation	[171]
	13-LOX-derived oxylipins	*Arabidopsis thaliana*	LOX6	Root-derived oxylipins contribute to drought resistance and systemic signaling	[168]
	GLVs	*Camellia sinensis*	Z-3-Hexenyl acetate	Enhances drought tolerance by activating phenylpropanoid metabolism	[170]
	PXG-derived oxylipins	*Arabidopsis thaliana*	RD20 (AtCLO3)	Regulates stomatal closure, transpiration and drought tolerance	[169]
Salinity	JA	*Ipomoea batatas*	JA biosynthesis and signaling genes	Salt stress induces JA signaling and JA-responsive genes associated with salt tolerance	[172]
	JA	*Arabidopsis thaliana*	LOX3, JA, MeJA	LOX3-mediated JA biosynthesis positively regulates salt tolerance; MeJA rescues the lox3 mutant phenotype	[173]
	JA	*Triticum aestivum*	TaAOC1, JA signaling	TaAOC1 enhances salt tolerance by increasing JA biosynthesis and activating JA-responsive genes	[174]
	JA	*Arabidopsis thaliana*	JA signaling pathway, primary root	Salt stress activates JA signaling, leading to inhibition of primary root cell elongation	[175]
	JA	*Oryza sativa*	OsOPR7	OsOPR7-mediated JA biosynthesis mitigates mitochondrial oxidative stress and enhances salt tolerance	[176]
	LOX-derived oxylipins (JA pathway)	*Glycine max*	Class II acyl-CoA-binding proteins (ACBPs), LOX	Ligand-dependent interaction between Class II ACBPs and LOX modulates oxylipin signaling and improves salt tolerance	[177]
	OPDA	*Oryza sativa*	AOC mutants, OPDA, ROS-scavenging enzymes	Reduced OPDA accumulation enhances salt tolerance through increased ROS-scavenging capacity	[86]
	OPDA	*Zea mays*	ZmEREB57, OPDA biosynthesis	ZmEREB57 promotes OPDA synthesis and enhances salt tolerance through two signaling pathways	[178]
	JA, OPDA	*Medicago truncatula*	Endogenous oxylipin profile	Salt stress dynamically remodels JA- and OPDA-related oxylipin metabolism	[179]
	PXG-derived oxylipins	*Oryza sativa*	OsPXG9, lipid hydroperoxides	Rice PXG catalyzes LOX-dependent epoxidation during abiotic stress responses, including drought and salinity	[28]
	PXG-derived oxylipins	*Oryza sativa*	OsClo5, OsDi19-5	OsClo5 negatively regulates salt tolerance through interaction with OsDi19-5	[180]
	GLVs	*Arachis hypogaea*	(Z)-3-Hexenyl acetate	GLV priming enhances salinity tolerance by improving antioxidant capacity and stress-responsive metabolism	[181]
Waterlogging or flooding	JA	*Cucumis sativus*	CsJAZ8, MYB6	CsJAZ8 interacts with MYB6 to regulate adventitious root formation during waterlogging	[182]
	JA	*Carthamus tinctorius*	CtMYB63	CtMYB63 enhances waterlogging tolerance through activation of JA signaling	[183]
	AOS and HPL-derived oxylipins	*Arabidopsis thaliana*	AOS and HPL pathway	Oxylipin-mediated metabolic reprogramming enhances waterlogging tolerance by maintaining energy metabolism and stress acclimation	[184]
Cold	JA	*Oryza sativa*	OsLPXC	OsLPXC negatively regulates cold tolerance by modulating JA accumulation, oxidative stress, and antioxidant defense	[185]
	JA	*Solanum lycopersicum*	MYB15, LOXD, MYC2	MYB15–LOXD and MYB15–MYC2–LOXD modules regulate JA signaling to enhance cold tolerance	[186]
	JA	*Arabidopsis thaliana*	Phytochrome A/B, ABA-dependent JA signaling	Phytochromes regulate cold tolerance through ABA-dependent JA signaling	[187]
	GLVs	*Zea mays*	GLVs	GLV pretreatment reduces chilling injury and improves seedling recovery	[188]
	KODA	*Oryza sativa*	KODA	Exogenous KODA promotes early rice growth under low-temperature conditions	[189]
Heat	JA	Wheat, *Arabidopsis*	HsfA1b, OPR3	HsfA1b promotes thermotolerance through OPR3-mediated JA signaling	[190]
	RES	*Marchantia polymorpha*, *Arabidopsis thaliana*	RES, HSFA1	RES enhance thermotolerance through an ancient COI1-independent signaling mechanism	[191]
	RES	*Arabidopsis thaliana*	HSFA1	RES activate HSFA1-dependent heat stress responses independently of JA perception	[100]
Heavy metals	JA	*Oryza sativa*	Auxin–JA signaling	Auxin–JA crosstalk regulates root system remodeling during Cd and/or As exposure	[192]
	OPDA	*Zygophyllum fabago*	OPDA, ABA	OPDA accumulation is associated with Pb tolerance and is enhanced by SA priming, indicating coordinated OPDA–ABA signaling during Pb stress	[193]
	MeJA	*Oryza sativa*	Exogenous MeJA	MeJA application alleviates arsenic toxicity by improving antioxidant defense and physiological performance	[194]
	MeJA	*Oryza sativa*	Exogenous MeJA	MeJA reduces Cd-induced oxidative damage and enhances antioxidant responses	[195]
UV-B	JA	*Arabidopsis thaliana*	UVR8–TCP4–LOX2	UVR8 activates TCP4-mediated LOX2 expression to promote UV-B tolerance	[196]
	LOX-derived oxylipins	*Arabidopsis thaliana*	UVR8, LOX1	UVR8 interacts with LOX1 to induce stomatal closure through LOX-derived oxylipin signaling	[197]
High light	JA	*Arabidopsis thaliana*	JA signaling, glutathione	JA signaling coordinates recovery from high-light stress through interaction with glutathione metabolism	[198]
	JA	*Populus*	MYC2–MYB113	MYC2–MYB113 module regulates anthocyanin accumulation and secondary wall thickening during high-light acclimation	[199]
	HPL-derived oxylipins	*Arabidopsis thaliana*	HPL pathway	HPL-derived oxylipins protect photosystems against photoinhibition during high-light stress	[200]
Drought + Cold	GLVs	*Camellia sinensis*	(Z)-3-Hexenol, ABA glucosylation pathway	(Z)-3-Hexenol enhances cold tolerance through ABA glucosylation	[201]
Heat + High light	JA	*Arabidopsis thaliana*	JA biosynthesis/signaling	JA is essential for acclimation to simultaneous high light and heat stress	[202]
Heat + Drought	JA	*Glycine max*	Exogenous JA	JA priming enhances antioxidant capacity, photosynthesis and tolerance under combined heat and drought stress	[203]
Heat + Cadmium	JA	*Arabidopsis thaliana*	JA-deficient mutants	Endogenous JA is required to mitigate damage caused by combined heat and cadmium stress	[204]
Salinity + UV-B	LOX-derived oxylipins	*Luffa acutangula*	Endogenous oxylipin metabolism	Oxylipin metabolism contributes to physiological adaptation under combined salinity and UV-B stress	[205]

#### 5.1.2. Salinity

Similar to drought, salinity stress triggers jasmonate-dependent signaling pathways; however, the relative abundance and biological functions of specific oxylipin pathways vary depending on the intensity and duration of the stress. Comparative metabolomic analyses have shown that salinity alters the endogenous oxylipin profile, leading to corresponding changes in metabolites derived from JA and OPDA [179]. JA biosynthesis is enhanced through the coordinated activity of LOX3, AOC, and OPR enzymes, resulting in the activation of salinity-responsive genes and an increase in antioxidant capacity [173,174,176]. During adaptation to salinity in soybean, ligand-dependent interactions between Class II ACBPs and LOX further regulate oxylipin-mediated signaling processes [177]. Nevertheless, JA signals also inhibit primary root cell elongation under saline conditions. This response demonstrates the balance between growth inhibition and stress tolerance [172,175].

Studies highlighted the contribution of other oxylipin classes to stress adaptation, such as rice OsPXG9 catalyzes LOX-dependent epoxidation of lipid hydroperoxides during drought and salinity [28], while the caleosin OsClo5 negatively regulates salt tolerance through interaction with OsDi19-5 [180]. In addition, priming with the GLV (Z)-3-hexenyl acetate enhances antioxidant capacity and metabolic adjustment under saline conditions in *Arachis hypogaea* [181]. But in terms of the role of OPDA under salinity, it appears to be more context-dependent than under drought stress. Reduced OPDA accumulation in AOC mutants was associated with enhanced ROS-scavenging capacity and improved salt tolerance in rice [86], while in maize, ZmEREB57 enhanced salt adaptation by promoting OPDA biosynthesis through two different signaling pathways [178]. These contrasting observations indicate that OPDA acts as a regulatory node, and its physiological outcome depends on species-specific signaling networks as well as the balance between stress signaling and oxidative defense processes.

#### 5.1.3. Flooding and Waterlogging

Flooding and soil waterlogging cause oxygen deficiency in plants, disrupting aerobic respiration and cellular energy metabolism. While oxylipin signals primarily coordinate water conservation and homeostasis under drought and salinity conditions, responses to flooding rely on changes that enable plants to adapt to hypoxic environments. Oxylipins contribute to this adaptation by integrating the remodeling of lipid composition with processes such as energy metabolism, antioxidant defense and developmental plasticity [206,207]. The remodeling of membrane lipids resulting from hypoxia implies changes in oxylipin biosynthesis, specifically, the involvement of lipid-derived signals in the transition from aerobic to anaerobic metabolism [208]. Among these, oxylipins generated via the HPL pathway play a crucial role. In *Arabidopsis thaliana*, activation of the HPL pathway facilitates metabolic reprogramming that preserves energy production and enhances adaptation to flooding [184]. JA signaling also contributes to flooding adaptation by regulating developmental responses. It has been reported that in cucumber, the CsJAZ8 protein interacts with the MYB6 transcription factor to control the formation of adventitious roots during flooding, while in safflower, the CtMYB63 factor enhances flooding tolerance by activating JA-dependent signaling [182,183].

### 5.2. Temperature-Related Stresses

#### 5.2.1. Cold

Cold-induced membrane remodelling has a direct impact on LOX activity and the biosynthesis of oxylipins since PUFAs serve as substrates for these enzymes. Preliminary studies in rice demonstrated that OsOPR1 is rapidly regulated in response to low temperatures [209]. Similarly, cold tolerance is closely linked to the maintenance of fatty acid unsaturation levels and LOX activity in figleaf gourd and cucumber, highlighting the importance of oxylipin production under chilling conditions [210]. According to recent findings, the MYB15–LOXD and MYB15–MYC2–LOXD regulatory modules stimulate JA biosynthesis and enhance cold tolerance by activating the expression of cold-responsive genes in tomato [186]. Conversely, OsLPXC acts as a negative regulator in rice. In this context, the suppression of JA accumulation reduces oxidative damage and enhances antioxidant defense during cold stress [185]. The crosstalk between light and hormonal signals further contributes to cold adaptation, as phytochromes regulate freezing tolerance through ABA-dependent JA signaling [187]. The involvement of other oxylipins in low-temperature responses has also been identified. As highlighted in the subsections on drought and salinity tolerance, pre-treating maize seedlings with GLVs also mitigates cold-induced damage and improves post-stress recovery [188]. A recent study indicates that the oxylipin KODA (9-hydroxy-10-oxo-12(Z),15(Z)-octadecadienoic acid) promotes early rice growth under low-temperature conditions [189].

#### 5.2.2. Heat

Heat stress promotes the peroxidation of membrane lipids, resulting in the rapid accumulation of different oxylipins, which contribute to heat tolerance via hormonal and redox-dependent signalling pathways (Table 2). Jasmonate signaling participates in heat adaptation by interacting with the heat shock response. In wheat and Arabidopsis, the heat shock TF HsfA1b enhances heat tolerance by activating OPR3-dependent JA biosynthesis. This process links oxylipin metabolism to heat-responsive transcriptional regulation [190]. Heat stress also triggers the release of GLVs, which increase with the intensity of the stress, demonstrating that volatile oxylipins contribute to both local and systemic adaptive responses [211]. Further research indicates that lipid-derived RES serve as an additional regulatory mechanism during heat stress. RES activates heat shock responses via signaling pathways that are dependent on HSFA1 but independent of COI1 [100,191]. These data summarize that heat and cold tolerance depend on the adaptive regulation of oxylipin metabolism, with specialized oxylipins acting cooperatively to optimize the process of adaptation to temperature stress [212,213].

### 5.3. Radiation- and Light-Related Stresses

#### 5.3.1. UV-B

UV-B radiation induces membrane lipid oxidation and oxylipin production, which activate signal transduction networks that protect plants from photooxidative damage. Early studies demonstrated that UV-B radiation leads to the accumulation of jasmonates and the activation of genes in the octadecanoid pathway [214,215]. Oxylipin signals generated in response to UV-B are closely linked to antioxidant defense, secondary metabolism, and photoprotective reactions, which collectively enhance plant tolerance to excessive radiation [216,217]. It has been recently reported that the perception of UV-B radiation is directly linked to oxylipin biosynthesis via the UVR8 signaling pathway. In *Arabidopsis thaliana*, activation of the UVR8–TCP4–LOX2 module stimulates jasmonate biosynthesis and enhances UV-B tolerance by linking photoreceptor signals to LOX-dependent oxylipin metabolism [196]. In addition to transcriptional regulation, UVR8 interacts with LOX1 to mediate stomatal closure via oxylipin signals generated by LOX. This indicates that oxylipins serve as a link between developmental and physiological responses to UV-B exposure [197].

#### 5.3.2. High Light

High-light exposure disrupts photosynthetic electron transport, leading to excessive ROS production and the oxidation of chloroplast membrane lipids. In addition to jasmonates, high light promotes the accumulation of chloroplast-derived oxylipins, which coordinate antioxidant responses, sulfur metabolism, and photoprotective acclimation [218,219]. JA regulates recovery from photoinhibition by coordinating antioxidant metabolism with glutathione-dependent redox regulation, thereby facilitating the restoration of photosynthetic activity following light stress [198]. High light also triggers secondary metabolic pathways via JA-responsive transcriptional networks. In *Populus*, the MYC2–MYB113 regulatory module links JA signaling to anthocyanin accumulation and secondary cell wall thickening, demonstrating how oxylipins contribute to both photoprotection and structural adaptation under prolonged light exposure [199]. At the same time, the HPL branch of the oxylipin pathway directly contributes to photoprotection by reducing photoinhibition and maintaining photosynthetic efficiency under excess light [200].

### 5.4. Heavy Metal Stress

Toxic metal exposure also significantly alters lipid oxidation patterns, leading to the fast remodelling of PUFAs within membranes during stress [220]. Changes in endogenous JA levels and phospholipid composition have been observed in various plant species exposed to cadmium, lead, and other heavy metals [221,222]. Recent reviews further emphasize that oxylipin signaling involved in regulating heavy metal tolerance also extensively interacts with antioxidant systems, sulfur metabolism, and hormonal pathways [223,224]. Studies have shown that the interplay between auxin and jasmonate signaling plays a vital role in regulating root system architecture in rice following exposure to cadmium and arsenic [192]. In *Zygophyllum fabago*, lead-induced stress triggered the accumulation of OPDA and ABA, as well as organ-specific metabolic changes. This, in turn, implies that OPDA participates in coordinating hormonal responses to metal toxicity [193]. Exogenous application of MeJA mitigates arsenic toxicity in rice and reduces cadmium-induced oxidative damage by enhancing antioxidant defense and maintaining membrane integrity [194,195]. Although the underlying molecular mechanisms remain less well characterized than for drought or salinity, existing evidence confirms the important role of oxylipin signaling under heavy metal stress.

### 5.5. Oxylipin Signaling Under Combined Abiotic Stresses

In natural environments, plants are often exposed to multiple abiotic stresses simultaneously. Combined stresses induce distinct physiological and metabolic responses that cannot be predicted from single-stress experiments, requiring the coordinated integration of multiple signaling pathways. Several studies indicate that jasmonate signaling is crucial for plant adaptation to combined environmental challenges. Under simultaneous high-light and heat stress, JA signaling promotes adaptation by coordinating antioxidant defense and maintaining photosynthetic performance [202]. Similarly, JA priming mitigates the detrimental effects of combined heat and drought stress in soybeans by enhancing antioxidant capacity and preserving photosynthetic activity [203]. (Z)-3-hexenol integrates stress responses via ABA-dependent metabolic regulation under combined drought and cold conditions [201]. Moreover, disruption of JA biosynthesis reduces tolerance to heat and cadmium stress, highlighting the importance of endogenous jasmonates in coordinating responses to simultaneous heat and heavy metal stresses [204]. Combined exposure to salinity and UV-B radiation triggers oxylipin-mediated antioxidant and physiological adaptations that enhance stress tolerance [205]. Although mechanistic studies are limited, existing evidence indicates that oxylipins function as signaling hubs integrating multiple environmental cues.

## 6. Knowledge Gaps and Future Perspectives

Despite significant advances in understanding the biosynthesis and signaling functions of plant oxylipins, several fundamental questions remain unresolved. While the canonical jasmonate pathway is relatively well-characterized, the perception and downstream signaling mechanisms of other emerging oxylipin pathways are not yet well understood. In particular, it is still unknown whether individual oxylipins act through specific receptors, bind directly to existing signaling proteins, or modify protein activity through their chemical properties. The functional diversity of non-JA oxylipins requires further attention, especially regarding the identification of distinct physiological effects under stress conditions. Addressing these questions is crucial for distinguishing prospective signaling molecules from metabolites that merely reflect membrane damage or oxidative stress.

One of the major challenges concerns network integration and signal specificity. Establishing causal relationships between oxylipin signals and integrated pathways remains difficult, as many studies only describe correlations in transcript or metabolite abundance without determining the sequence of signaling events. Thus, future research needs to move beyond single-pathway analyses toward perturbation-based approaches that monitor multiple signaling layers simultaneously. Oxylipin dynamics also require more precise characterization under combined stress conditions and across various developmental stages.

Finally, greater emphasis should be placed on comparative and translational research. Oxylipin signaling components can vary significantly among plant species, and mechanisms established in Arabidopsis cannot be directly extrapolated to other crops. Integrating comparative genomics, lipidomics, genetic perturbation, and quantitative modeling across crop species could reveal conserved signaling modules as well as species-specific regulatory mechanisms.

## 7. Conclusions

Oxylipin signalling is now widely recognized as a complex, integrated regulatory system rather than a single jasmonate-centered pathway. While the COI1–JAZ–MYC module provides the best-resolved signaling framework, other oxylipins contribute through diverse and interconnected mechanisms involving redox status, Ca^2+^ dynamics, MAPKs, ABA, and stress-responsive transcriptional networks. Their effects are highly dependent on the oxylipin class, tissue, developmental context, and abiotic stress conditions. A major priority for future research is therefore to define how these chemically diverse lipid signals are integrated within cellular networks and translated into appropriate adaptive responses. Such knowledge will be essential for determining which oxylipin pathways can be selectively manipulated to enhance crop resilience under abiotic stresses.

## Figures and Tables

**Table 1:** Molecular crosstalk between oxylipin classes and cellular signaling networks during plant abiotic stress responses.

Oxylipin Class	Primary Signaling Networks	Key Signaling Components	Major Molecular Mechanism of Crosstalk	Representative Downstream Regulators	Representative Experimental Species	Evidence Context
**Jasmonates (JA, JA-Ile)**	ROS, Ca^2+^, MAPKs, ABA, ET, SA, BR, Auxin, GA	COI1, JAZ, MYC2, MPK3/6, SnRK2, EIN3, DELLA, BZR1	COI1-dependent receptor signaling, ubiquitin-mediated JAZ degradation, phosphorylation cascades, transcriptional integration	MYC2, WRKY, NAC, DREB, ERF, ABI5, BZR1	*Arabidopsis*, rice, pepper, tomato	Genetic/mutant, biochemical, and transcriptomic studies
**OPDA**	ROS, Ca^2+^, MAPKs, ABA	CYP20-3, glutathione, chloroplast redox system	COI1-independent electrophilic/redox signaling; chloroplast-to-nucleus communication	TGA, WRKY, NAC	*Arabidopsis*, rice	Genetic/mutant, redox and signaling studies
**GLVs**	Ca^2+^, MAPKs, ET	GLRs/CNGCs (putative), MPK3/6, WRKY6, WRKY40	Rapid Ca^2+^ influx, MAPK phosphorylation, volatile-mediated signaling, transcriptional priming	WRKY6, WRKY40, ERFs	*Arabidopsis*, *Lolium*, maize	Volatile exposure, Ca^2+^ imaging, MAPK activation and transcriptional/mutant studies
**PhytoPs**	ROS, MAPKs	MAPKs, ROS, GST-related responses	ROS-derived signaling; MAPK activation; regioisomer-specific transcription	PAL, Lin6, defense-associated genes	tomato, *Arabidopsis*	Exogenous application, MAPK activation, and defense-response assays
**PXG-derived oxylipins**	ROS, ABA, GA	RD20 (AtCLO3), AtCLO4, CaJAZ1-03, CaASRF1	Peroxygenase-mediated lipid peroxide metabolism; hormone-associated regulation	ABF3, ABF4	*Arabidopsis*, rice	Biochemical enzyme characterization and genetic/functional studies

## Data Availability

No new data were created or analyzed in this study. Data sharing is not applicable to this article.

## Abbreviations

The following abbreviations are used in this manuscript:

JA	jasmonic acid
OPDA	12-oxo-phytodienoic acid
GLVs	green leaf volatiles
RES	reactive electrophile species
PXG	peroxygenase
ROS	reactive oxygen species
Ca^2+^	Calcium ions
MAPK	mitogen-activated protein kinase
UV	ultraviolet
PUFA	polyunsaturated fatty acid
PhytoPs	phytoprostanes
LOX	lipoxygenase
ABA	abscisic acid
SA	salicylic acid
BRs	brassinosteroids
LnA	linolenic acid
LA	linoleic acid
HTA	hexadecatrienoic acid
MGDG	monogalactosyldiacylglycerol
FAHPs	fatty acid hydroperoxides
13-HPOT	13-hydroperoxy-α-linolenic acid
AOS	allene oxide synthase
AOC	allene oxide cyclase
JAR1	Jasmonate resistant 1
JA-Ile	jasmonoyl-isoleucine
COI1	CORONATINE INSENSITIVE 1
JAZ	JASMONATE ZIM-DOMAIN
KODA	9-hydroxy-10-oxo-12(Z),15(Z)-octadecadienoic acid
HPL	hydroperoxide lyase
DES	divinyl ether synthase
dn-OPDA	dinor-OPDA
α-DOX	α-dioxygenase
OPR3	12-oxophytodienoate reductase 3
CYP20-3	cyclophilin 20-3
TFs	transcription factors
miRNA	microRNA
ABF	ABA-responsive element-binding factor
AREB	ABA-responsive element-binding protein
CBF	C-repeat binding factor
DREB	dehydration-responsive element-binding protein
ERFs	ethylene response factors
HSFA1	heat shock factor A1
MYC	MYC family basic helix–loop–helix transcription factors
NAC	NAM/ATAF/CUC transcription factors
WRKY	WRKY transcription factors
PTMs	Post-Translational Modification
SCF	SKP1–Cullin1–F-box
NINJA	Novel Interactor of JAZ
TPL	TOPLESS
EAR	Ethylene-responsive element-binding factor-associated Amphiphilic Repression
bHLH	basic helix–loop–helix
HAC1	histone acetyltransferase
MED25	Mediator of RNA polymerase II transcription subunit 25
tn-OPDA	tetranor-cis-OPDA
4,5-ddh-JA	7-iso-4,5-didehydro-jasmonic acid
GSH	reduced glutathione
bZIP	basic leucine zipper
TCP	Teosinte branched1/Cycloidea/Pcf
RBOH	Respiratory Burst Oxidase Homologs
SOD	superoxide dismutase
CAT	catalase
APX	ascorbate peroxidase
GPX	glutathione peroxidases
GST	glutathione S-transferases
GLRs	glutamate receptor-like channels
CNGCs	cyclic nucleotide-gated channels
OSCAs	osmosensitive calcium-permeable channels
CaMs	calmodulins
CMLs	calmodulin-like proteins
CDPKs/CPKs	calcium-dependent protein kinases
CBL	calcineurin B-like protein
CIPK	CBL-interacting protein kinase
MAPKKs	MAPK kinases
MAPKKKs	MAPK kinase kinases
MeJA	methyl jasmonate
DAMPs	damage-associated molecular patterns
BRI1	Brassinosteroid insensitive1
BAK1	BRI1-associated receptor kinase 1
BIN2	Brassinosteroid-insensitive 2
PLT	PLETHORA
ARF	Auxin response factor
PIN	PIN-FORMED
RD20	Responsive to desiccation20
ACBPs	Acyl-CoA-binding proteins

## References

[1] Boyer J.S. (1982). Plant Productivity and Environment. Science.

[2] Bailey-Serres J., Voesenek L.A.C.J. (2008). Flooding Stress: Acclimations and Genetic Diversity. Annu. Rev. Plant Biol..

[3] Lamaoui M., Jemo M., Datla R., Bekkaoui F. (2018). Heat and Drought Stresses in Crops and Approaches for Their Mitigation. Front. Chem..

[4] Zandalinas S.I., Fritschi F.B., Mittler R. (2021). Global Warming, Climate Change, and Environmental Pollution: Recipe for a Multifactorial Stress Combination Disaster. Trends Plant Sci..

[5] Lee H., Romero J. (2024). Climate Change 2023 Synthesis Report. Contribution of Working Groups I, II and III to the Sixth Assessment Report of the Intergovernmental Panel on Climate Change.

[6] Ortiz-Bobea A., Ault T.R., Carrillo C.M., Chambers R.G., Lobell D.B. (2021). Anthropogenic Climate Change Has Slowed Global Agricultural Productivity Growth. Nat. Clim. Change.

[7] Verma V., Ravindran P., Kumar P.P. (2016). Plant Hormone-Mediated Regulation of Stress Responses. BMC Plant Biol..

[8] Thilakarathne A.S., Liu F., Zou Z. (2025). Plant Signaling Hormones and Transcription Factors: Key Regulators of Plant Responses to Growth, Development, and Stress. Plants.

[9] Feussner I., Wasternack C. (2002). The lipoxygenase pathway. Annu. Rev. Plant Biol..

[10] Pérez-Llorca M., Pollmann S., Müller M. (2023). Ethylene and Jasmonates Signaling Network Mediating Secondary Metabolites under Abiotic Stress. Int. J. Mol. Sci..

[11] Kuźniak E., Gajewska E. (2024). Lipids and Lipid-Mediated Signaling in Plant–Pathogen Interactions. Int. J. Mol. Sci..

[12] Lim G.-H., Singhal R., Kachroo A., Kachroo P. (2017). Fatty Acid– and Lipid-Mediated Signaling in Plant Defense. Annu. Rev. Phytopathol..

[13] Heilmann I. (2016). Plant Phosphoinositide Signaling—Dynamics on Demand. Biochim. Biophys. Acta (BBA)—Mol. Cell Biol. Lipids.

[14] Agrawal G.K., Tamogami S., Han O., Iwahashi H., Rakwal R. (2004). Rice Octadecanoid Pathway. Biochem. Biophys. Res. Commun..

[15] Eckardt N.A. (2008). Oxylipin Signaling in Plant Stress Responses. Plant Cell.

[16] Mosblech A., Feussner I., Heilmann I. (2009). Oxylipins: Structurally Diverse Metabolites from Fatty Acid Oxidation. Plant Physiol. Biochem..

[17] Wasternack C., Feussner I. (2018). The Oxylipin Pathways: Biochemistry and Function. Annu. Rev. Plant Biol..

[18] Matsui K., Sugimoto K., Kakumyan P., Khorobrykh S.A., Mano J., Armstrong D. (2009). Volatile Oxylipins and Related Compounds Formed Under Stress in Plants. Lipidomics.

[19] Griffiths G. (2015). Biosynthesis and Analysis of Plant Oxylipins. Free Radic. Res..

[20] Liechti R., Farmer E.E. (2002). The Jasmonate Pathway. Science.

[21] Blée E. (2002). Impact of Phyto-Oxylipins in Plant Defense. Trends Plant Sci..

[22] Reinbothe C., Springer A., Samol I., Reinbothe S. (2009). Plant Oxylipins: Role of Jasmonic Acid during Programmed Cell Death, Defence and Leaf Senescence. FEBS J..

[23] Kim E.-S., Choi E., Kim Y., Cho K., Lee A., Shim J., Rakwal R., Agrawal G.K., Han O. (2003). Dual Positional Specificity and Expression of Non-Traditional Lipoxygenase Induced by Wounding and Methyl Jasmonate in Maize Seedlings. Plant Mol. Biol..

[24] Wasternack C., Hause B. (2013). Jasmonates: Biosynthesis, Perception, Signal Transduction and Action in Plant Stress Response, Growth and Development. An Update to the 2007 Review in Annals of Botany. Ann. Bot..

[25] Knieper M., Viehhauser A., Dietz K.-J. (2023). Oxylipins and Reactive Carbonyls as Regulators of the Plant Redox and Reactive Oxygen Species Network under Stress. Antioxidants.

[26] Dubery I.A. (2026). Arabidopsides as Signatory Biomarkers of the *Arabidopsis thaliana* Response to Lipopolysaccharides—Metabolomics Insights. Int. J. Mol. Sci..

[27] Tran A.D., Cho K., Vu M.A., Kim J.-I., Nguyen H.T.T., Han O. (2025). Rice Peroxygenase-9 Negatively Regulates Production of Reactive Oxygen Species and Increases Cellular Resistance to Abiotic Stress. Int. J. Mol. Sci..

[28] Tran A.D., Cho K., Han O. (2023). Rice Peroxygenase Catalyzes Lipoxygenase-Dependent Regiospecific Epoxidation of Lipid Peroxides in the Response to Abiotic Stressors. Bioorg. Chem..

[29] Mueller M.J. (2004). Archetype Signals in Plants: The Phytoprostanes. Curr. Opin. Plant Biol..

[30] Durand T., Bultel-Poncé V., Guy A., Berger S., Mueller M.J., Galano J. (2009). New Bioactive Oxylipins Formed by Non-Enzymatic Free-Radical-Catalyzed Pathways: The Phytoprostanes. Lipids.

[31] Mersni M., Zhou B., Reversat G., Khouja M.L., Guy A., Oger C., Galano J.-M., Durand T., Messaoud C., Vigor C. (2024). Phytoprostanes and Phytofurans: Bioactive Compounds in Aerial Parts of Acacia Cyanophylla Lindl. Fitoterapia.

[32] Engelberth J. (2024). Green Leaf Volatiles: A New Player in the Protection against Abiotic Stresses?. Int. J. Mol. Sci..

[33] Liu H., Timko M.P. (2021). Jasmonic Acid Signaling and Molecular Crosstalk with Other Phytohormones. Int. J. Mol. Sci..

[34] Yu Q., Hua X., Yao H., Zhang Q., He J., Peng L., Li D., Yang Y., Li X. (2021). Abscisic Acid Receptors Are Involves in the Jasmonate Signaling in *Arabidopsis*. Plant Signal. Behav..

[35] Bukhonska Y., Derevyanchuk M., Filepova R., Martinec J., Dobrev P., Ruelland E., Kravets V. (2025). Brassinosteroid Synthesis and Perception Differently Regulate Phytohormone Networks in *Arabidopsis thaliana*. Int. J. Mol. Sci..

[36] Ameye M., Allmann S., Verwaeren J., Smagghe G., Haesaert G., Schuurink R.C., Audenaert K. (2018). Green Leaf Volatile Production by Plants: A Meta-analysis. New Phytol..

[37] Osipova E.V., Lantsova N.V., Chechetkin I.R., Mukhitova F.K., Hamberg M., Grechkin A.N. (2010). Hexadecanoid Pathway in Plants: Lipoxygenase Dioxygenation of (7Z,10Z,13Z)-Hexadecatrienoic Acid. Biochemistry.

[38] Upchurch R.G. (2008). Fatty Acid Unsaturation, Mobilization, and Regulation in the Response of Plants to Stress. Biotechnol. Lett..

[39] Yoeun S., Sukhanov A., Han O. (2016). Separation of Enzymatic Functions and Variation of Spin State of Rice Allene Oxide Synthase-1 by Mutation of Phe-92 and Pro-430. Bioorg. Chem..

[40] Wasternack C., Strnad M. (2019). Jasmonates Are Signals in the Biosynthesis of Secondary Metabolites—Pathways, Transcription Factors and Applied Aspects—A Brief Review. New Biotechnol..

[41] Sheard L.B., Tan X., Mao H., Withers J., Ben-Nissan G., Hinds T.R., Kobayashi Y., Hsu F.-F., Sharon M., Browse J. (2010). Jasmonate Perception by Inositol-Phosphate-Potentiated COI1–JAZ Co-Receptor. Nature.

[42] Berg-Falloure K.M., Kolomiets M.V. (2023). Ketols Emerge as Potent Oxylipin Signals Regulating Diverse Physiological Processes in Plants. Plants.

[43] Jimenez-Aleman G.H., Jander G. (2023). Maize Defense against Insect Herbivory: A Novel Role for 9-LOX-Derived Oxylipins. Mol. Plant.

[44] Meesapyodsuk D., Qiu X. (2011). A Peroxygenase Pathway Involved in the Biosynthesis of Epoxy Fatty Acids in Oat. Plant Physiol..

[45] Andersson M.X., Hamberg M., Kourtchenko O., Brunnström Å., McPhail K.L., Gerwick W.H., Göbel C., Feussner I., Ellerström M. (2006). Oxylipin Profiling of the Hypersensitive Response in *Arabidopsis thaliana*: Formation of a novel oxo-phytodienoic acid-containing galactolipid, arabidopside E. J. Biol. Chem..

[46] Böttcher C., Pollmann S. (2009). Plant Oxylipins: Plant Responses to 12-oxo-phytodienoic Acid Are Governed by Its Specific Structural and Functional Properties. FEBS J..

[47] Sanz A., Moreno J.I., Castresana C. (1998). PIOX, a New Pathogen-Induced Oxygenase with Homology to Animal Cyclooxygenase. Plant Cell.

[48] Bannenberg G., Martínez M., Rodríguez M.J., López M.A., Ponce De León I., Hamberg M., Castresana C. (2009). Functional Analysis of *α*-DOX2, an Active *α*-Dioxygenase Critical for Normal Development in Tomato Plants. Plant Physiol..

[49] Farmer E.E., Mueller M.J. (2013). ROS-Mediated Lipid Peroxidation and RES-Activated Signaling. Annu. Rev. Plant Biol..

[50] Pinciroli M., Domínguez-Perles R., Garbi M., Abellán A., Oger C., Durand T., Galano J.M., Ferreres F., Gil-Izquierdo A. (2018). Impact of Salicylic Acid Content and Growing Environment on Phytoprostane and Phytofuran (Stress Biomarkers) in *Oryza sativa* L.. J. Agric. Food Chem..

[51] Savchenko T.V., Zastrijnaja O.M., Klimov V.V. (2014). Oxylipins and Plant Abiotic Stress Resistance. Biochemistry.

[52] Wang Y., Mostafa S., Zeng W., Jin B. (2021). Function and Mechanism of Jasmonic Acid in Plant Responses to Abiotic and Biotic Stresses. Int. J. Mol. Sci..

[53] Hanano A., Blée E., Murphy D.J. (2023). Caleosin/Peroxygenases: Multifunctional Proteins in Plants. Ann. Bot..

[54] Yi R., Li Y., Shan X. (2024). OPDA/Dn-OPDA Actions: Biosynthesis, Metabolism, and Signaling. Plant Cell Rep..

[55] Park S.-W., Li W., Viehhauser A., He B., Kim S., Nilsson A.K., Andersson M.X., Kittle J.D., Ambavaram M.M.R., Luan S. (2013). Cyclophilin 20-3 Relays a 12-Oxo-Phytodienoic Acid Signal during Stress Responsive Regulation of Cellular Redox Homeostasis. Proc. Natl. Acad. Sci. USA.

[56] Scala A., Allmann S., Mirabella R., Haring M., Schuurink R. (2013). Green Leaf Volatiles: A Plant’s Multifunctional Weapon against Herbivores and Pathogens. Int. J. Mol. Sci..

[57] Findling S., Stotz H.U., Zoeller M., Krischke M., Zander M., Gatz C., Berger S., Mueller M.J. (2018). TGA2 Signaling in Response to Reactive Electrophile Species Is Not Dependent on Cysteine Modification of TGA2. PLoS ONE.

[58] Lange M., Korte A., Fuchs M., Fekete A., Mueller C., Dierich B., Witte J., Dandekar T., Mueller M.J., Berger S. (2025). A CW-Type Zinc Finger Protein Is Involved in RES-Oxylipin Signaling and the Response to Abiotic Stress in *Arabidopsis thaliana*. Front. Plant Sci..

[59] Genva M., Obounou Akong F., Andersson M.X., Deleu M., Lins L., Fauconnier M.-L. (2019). New Insights into the Biosynthesis of Esterified Oxylipins and Their Involvement in Plant Defense and Developmental Mechanisms. Phytochem. Rev..

[60] Steppuhn A., Gaquerel E., Baldwin I.T. (2010). The Two α-Dox Genes of *Nicotiana attenuata*: Overlapping but Distinct Functions in Development and Stress Responses. BMC Plant Biol..

[61] Farmer E.E. (2007). Jasmonate Perception Machines. Nature.

[62] Wasternack C., Song S. (2017). Jasmonates: Biosynthesis, Metabolism, and Signaling by Proteins Activating and Repressing Transcription. J. Exp. Bot..

[63] Hu Y., Jiang Y., Han X., Wang H., Pan J., Yu D. (2017). Jasmonate Regulates Leaf Senescence and Tolerance to Cold Stress: Crosstalk with Other Phytohormones. J. Exp. Bot..

[64] Shikha D., Jakhar P., Satbhai S.B. (2023). Role of Jasmonate Signaling in the Regulation of Plant Responses to Nutrient Deficiency. J. Exp. Bot..

[65] Chini A., Fonseca S., Fernández G., Adie B., Chico J.M., Lorenzo O., García-Casado G., López-Vidriero I., Lozano F.M., Ponce M.R. (2007). The JAZ Family of Repressors Is the Missing Link in Jasmonate Signalling. Nature.

[66] Thines B., Katsir L., Melotto M., Niu Y., Mandaokar A., Liu G., Nomura K., He S.Y., Howe G.A., Browse J. (2007). JAZ Repressor Proteins Are Targets of the SCFCOI1 Complex during Jasmonate Signalling. Nature.

[67] Pauwels L., Barbero G.F., Geerinck J., Tilleman S., Grunewald W., Pérez A.C., Chico J.M., Bossche R.V., Sewell J., Gil E. (2010). NINJA Connects the Co-Repressor TOPLESS to Jasmonate Signalling. Nature.

[68] Lorenzo O., Chico J.M., Sánchez-Serrano J.J., Solano R. (2004). *JASMONATE-INSENSITIVE1* Encodes a MYC Transcription Factor Essential to Discriminate between Different Jasmonate-Regulated Defense Responses in Arabidopsis. Plant Cell.

[69] Kazan K., Manners J.M. (2013). MYC2: The Master in Action. Mol. Plant.

[70] Fernández-Calvo P., Chini A., Fernández-Barbero G., Chico J.-M., Gimenez-Ibanez S., Geerinck J., Eeckhout D., Schweizer F., Godoy M., Franco-Zorrilla J.M. (2011). The *Arabidopsis* bHLH Transcription Factors MYC3 and MYC4 Are Targets of JAZ Repressors and Act Additively with MYC2 in the Activation of Jasmonate Responses. Plant Cell.

[71] Pauwels L., Goossens A. (2011). The JAZ Proteins: A Crucial Interface in the Jasmonate Signaling Cascade. Plant Cell.

[72] Howe G.A., Major I.T., Koo A.J. (2018). Modularity in Jasmonate Signaling for Multistress Resilience. Annu. Rev. Plant Biol..

[73] Çevik V., Kidd B.N., Zhang P., Hill C., Kiddle S., Denby K.J., Holub E.B., Cahill D.M., Manners J.M., Schenk P.M. (2012). MEDIATOR25 Acts as an Integrative Hub for the Regulation of Jasmonate-Responsive Gene Expression in Arabidopsis. Plant Physiol..

[74] Zhai Q., Deng L., Li C. (2020). Mediator Subunit MED25: At the Nexus of Jasmonate Signaling. Curr. Opin. Plant Biol..

[75] Chen R., Jiang H., Li L., Zhai Q., Qi L., Zhou W., Liu X., Li H., Zheng W., Sun J. (2012). The *Arabidopsis* Mediator Subunit MED25 Differentially Regulates Jasmonate and Abscisic Acid Signaling through Interacting with the MYC2 and ABI5 Transcription Factors. Plant Cell.

[76] Zander M., Lewsey M.G., Clark N.M., Yin L., Bartlett A., Saldierna Guzmán J.P., Hann E., Langford A.E., Jow B., Wise A. (2020). Integrated Multi-Omics Framework of the Plant Response to Jasmonic Acid. Nat. Plants.

[77] Stintzi A., Weber H., Reymond P., Browse J., Farmer E.E. (2001). Plant Defense in the Absence of Jasmonic Acid: The Role of Cyclopentenones. Proc. Natl. Acad. Sci. USA.

[78] Taki N., Sasaki-Sekimoto Y., Obayashi T., Kikuta A., Kobayashi K., Ainai T., Yagi K., Sakurai N., Suzuki H., Masuda T. (2005). 12-Oxo-Phytodienoic Acid Triggers Expression of a Distinct Set of Genes and Plays a Role in Wound-Induced Gene Expression in *Arabidopsis*. Plant Physiol..

[79] Dave A., Hernández M.L., He Z., Andriotis V.M.E., Vaistij F.E., Larson T.R., Graham I.A. (2011). 12-Oxo-Phytodienoic Acid Accumulation during Seed Development Represses Seed Germination in *Arabidopsis*. Plant Cell.

[80] Ueda M., Saito R., Nishizato Y., Kitajima T., Kato N. (2025). Downstream Metabolites of (+)-Cis-12-Oxo-Phytodienoic Acid Function as Noncanonical Bioactive Jasmonates in *Arabidopsis thaliana*. Nat. Commun..

[81] Mekkaoui K., Baral R., Smith F., Klein M., Feussner I., Hause B. (2025). Transcriptomics and Trans-Organellar Complementation Reveal Limited Signaling of 12-Cis-Oxo-Phytodienoic Acid during Early Wound Response in *Arabidopsis*. Nat. Commun..

[82] Resemann H.C., Feussner K., Hornung E., Feussner I. (2023). A Non-Targeted Metabolomics Analysis Identifies Wound-Induced Oxylipins in Physcomitrium Patens. Front. Plant Sci..

[83] Liu S., Li T., Zhang P., Zhao L., Yi D., Zhang Z., Cong B. (2022). Insights into the Jasmonate Signaling in Basal Land Plant Revealed by the Multi-Omics Analysis of an Antarctic Moss *Pohlia nutans* Treated with OPDA. Int. J. Mol. Sci..

[84] Mueller S., Hilbert B., Dueckershoff K., Roitsch T., Krischke M., Mueller M.J., Berger S. (2008). General Detoxification and Stress Responses Are Mediated by Oxidized Lipids through TGA Transcription Factors in *Arabidopsis*. Plant Cell.

[85] Adhikari A., Park S.-W. (2023). Reduced GSH Acts as a Metabolic Cue of OPDA Signaling in Coregulating Photosynthesis and Defense Activation under Stress. Plants.

[86] Hazman M., Hause B., Eiche E., Nick P., Riemann M. (2015). Increased Tolerance to Salt Stress in OPDA-Deficient Rice Allene oxide cyclase Mutants Is Linked to an Increased ROS-Scavenging Activity. J. Exp. Bot..

[87] Curaba J., Singh M.B., Bhalla P.L. (2014). miRNAs in the Crosstalk between Phytohormone Signalling Pathways. J. Exp. Bot..

[88] Schommer C., Palatnik J.F., Aggarwal P., Chételat A., Cubas P., Farmer E.E., Nath U., Weigel D. (2008). Control of Jasmonate Biosynthesis and Senescence by miR319 Targets. PLoS Biol..

[89] Zhou M., Li D., Li Z., Hu Q., Yang C., Zhu L., Luo H. (2013). Constitutive Expression of a *miR319* Gene Alters Plant Development and Enhances Salt and Drought Tolerance in Transgenic Creeping Bentgrass. Plant Physiol..

[90] Ren L., Zhang T., Wu H., Ge X., Wan H., Chen S., Li Z., Ma D., Wang A. (2022). Blocking IbmiR319a Impacts Plant Architecture and Reduces Drought Tolerance in Sweet Potato. Genes.

[91] Shamloo-Dashtpagerdi R., Shahriari A.G., Tahmasebi A., Vetukuri R.R. (2023). Potential Role of the Regulatory miR1119-MYC2 Module in Wheat (*Triticum aestivum* L.) Drought Tolerance. Front. Plant Sci..

[92] Mirabella R., Rauwerda H., Allmann S., Scala A., Spyropoulou E.A., De Vries M., Boersma M.R., Breit T.M., Haring M.A., Schuurink R.C. (2015). WRKY40 and WRKY6 Act Downstream of the Green Leaf Volatile *E*-2-hexenal in *Arabidopsis*. Plant J..

[93] Staiger D., Brown J.W.S. (2013). Alternative Splicing at the Intersection of Biological Timing, Development, and Stress Responses. Plant Cell.

[94] Laloum T., Martín G., Duque P. (2018). Alternative Splicing Control of Abiotic Stress Responses. Trends Plant Sci..

[95] Chung H.S., Cooke T.F., DePew C.L., Patel L.C., Ogawa N., Kobayashi Y., Howe G.A. (2010). Alternative Splicing Expands the Repertoire of Dominant JAZ Repressors of Jasmonate Signaling. Plant J..

[96] Moreno J.E., Shyu C., Campos M.L., Patel L.C., Chung H.S., Yao J., He S.Y., Howe G.A. (2013). Negative Feedback Control of Jasmonate Signaling by an Alternative Splice Variant of JAZ10. Plant Physiol..

[97] Wu S., Hu C., Zhu C., Fan Y., Zhou J., Xia X., Shi K., Zhou Y., Foyer C.H., Yu J. (2024). The MYC2–PUB22–JAZ4 Module Plays a Crucial Role in Jasmonate Signaling in Tomato. Mol. Plant.

[98] Chico J.M., Lechner E., Fernandez-Barbero G., Canibano E., García-Casado G., Franco-Zorrilla J.M., Hammann P., Zamarreño A.M., García-Mina J.M., Rubio V. (2020). CUL3^BPM^ E3 Ubiquitin Ligases Regulate MYC2, MYC3, and MYC4 Stability and JA Responses. Proc. Natl. Acad. Sci. USA.

[99] Knieper M., Vogelsang L., Guntelmann T., Sproß J., Gröger H., Viehhauser A., Dietz K.-J. (2022). OPDAylation of Thiols of the Redox Regulatory Network In vitro. Antioxidants.

[100] Muench M., Hsin C.-H., Ferber E., Berger S., Mueller M.J. (2016). Reactive Electrophilic Oxylipins Trigger a Heat Stress-like Response through HSFA1 Transcription Factors. J. Exp. Bot..

[101] Mittler R., Vanderauwera S., Suzuki N., Miller G., Tognetti V.B., Vandepoele K., Gollery M., Shulaev V., Van Breusegem F. (2011). ROS Signaling: The New Wave?. Trends Plant Sci..

[102] Choudhury F.K., Rivero R.M., Blumwald E., Mittler R. (2017). Reactive Oxygen Species, Abiotic Stress and Stress Combination. Plant J..

[103] Mueller M.J., Berger S. (2009). Reactive Electrophilic Oxylipins: Pattern Recognition and Signalling. Phytochemistry.

[104] Higdon A., Diers A.R., Oh J.Y., Landar A., Darley-Usmar V.M. (2012). Cell Signalling by Reactive Lipid Species: New Concepts and Molecular Mechanisms. Biochem. J..

[105] Parvez S., Long M.J.C., Poganik J.R., Aye Y. (2018). Redox Signaling by Reactive Electrophiles and Oxidants. Chem. Rev..

[106] Kadota Y., Shirasu K., Zipfel C. (2015). Regulation of the NADPH Oxidase RBOHD During Plant Immunity. Plant Cell Physiol..

[107] Fichman Y., Mittler R. (2020). Rapid Systemic Signaling during Abiotic and Biotic Stresses: Is the ROS Wave Master of All Trades?. Plant J..

[108] Maruta T., Inoue T., Tamoi M., Yabuta Y., Yoshimura K., Ishikawa T., Shigeoka S. (2011). *Arabidopsis* NADPH Oxidases, AtrbohD and AtrbohF, Are Essential for Jasmonic Acid-Induced Expression of Genes Regulated by MYC2 Transcription Factor. Plant Sci..

[109] Devireddy A.R., Arbogast J., Mittler R. (2020). Coordinated and Rapid Whole-plant Systemic Stomatal Responses. New Phytol..

[110] Zhan C., Xue N., Su Z., Zheng T., Wu J. (2025). RBOHD, GLR3.3, and GLR3.6 Cooperatively Control Wounding Hypocotyl-Induced Systemic Ca^2+^ Signals, Jasmonic Acid, and Glucosinolates in Arabidopsis Leaves. Plant Divers..

[111] Knieper M., Schwarz R., Vogelsang L., Sproß J., Kaya A., Bittmann M., Gröger H., Viehhauser A., Dietz K. (2026). The Competitive Interplay of 12-oxophytodienoic Acid (OPDA), Protein Thiols, and Glutathione. FEBS J..

[112] Foyer C.H., Noctor G. (2005). Oxidant and Antioxidant Signalling in Plants: A Re-evaluation of the Concept of Oxidative Stress in a Physiological Context. Plant Cell Environ..

[113] Waszczak C., Carmody M., Kangasjärvi J. (2018). Reactive Oxygen Species in Plant Signaling. Annu. Rev. Plant Biol..

[114] Chandru H.K., Kim E., Kuk Y., Cho K., Han O. (2003). Kinetics of Wound-Induced Activation of Antioxidative Enzymes in *Oryza sativa*: Differential Activation at Different Growth Stages. Plant Sci..

[115] Mittler R., Vanderauwera S., Gollery M., Van Breusegem F. (2004). Reactive Oxygen Gene Network of Plants. Trends Plant Sci..

[116] Gill S.S., Tuteja N. (2010). Reactive Oxygen Species and Antioxidant Machinery in Abiotic Stress Tolerance in Crop Plants. Plant Physiol. Biochem..

[117] Cho K., Kim Y.C., Woo J.C., Rakwal R., Agrawal G.K., Yoeun S., Han O. (2012). Transgenic Expression of Dual Positional Maize Lipoxygenase-1 Leads to the Regulation of Defense-Related Signaling Molecules and Activation of the Antioxidative Enzyme System in Rice. Plant Sci..

[118] Dodd A.N., Kudla J., Sanders D. (2010). The Language of Calcium Signaling. Annu. Rev. Plant Biol..

[119] Kudla J., Becker D., Grill E., Hedrich R., Hippler M., Kummer U., Parniske M., Romeis T., Schumacher K. (2018). Advances and Current Challenges in Calcium Signaling. New Phytol..

[120] Yuan F., Yang H., Xue Y., Kong D., Ye R., Li C., Zhang J., Theprungsirikul L., Shrift T., Krichilsky B. (2014). OSCA1 Mediates Osmotic-Stress-Evoked Ca^2+^ Increases Vital for Osmosensing in *Arabidopsis*. Nature.

[121] Toyota M., Spencer D., Sawai-Toyota S., Jiaqi W., Zhang T., Koo A.J., Howe G.A., Gilroy S. (2018). Glutamate Triggers Long-Distance, Calcium-Based Plant Defense Signaling. Science.

[122] Wang X. (2004). Lipid Signaling. Curr. Opin. Plant Biol..

[123] Aratani Y., Uemura T., Hagihara T., Matsui K., Toyota M. (2023). Green Leaf Volatile Sensory Calcium Transduction in *Arabidopsis*. Nat. Commun..

[124] Boudsocq M., Sheen J. (2013). CDPKs in Immune and Stress Signaling. Trends Plant Sci..

[125] Tang R.-J., Wang C., Li K., Luan S. (2020). The CBL–CIPK Calcium Signaling Network: Unified Paradigm from 20 Years of Discoveries. Trends Plant Sci..

[126] Förster S., Schmidt L.K., Kopic E., Anschütz U., Huang S., Schlücking K., Köster P., Waadt R., Larrieu A., Batistič O. (2019). Wounding-Induced Stomatal Closure Requires Jasmonate-Mediated Activation of GORK K+ Channels by a Ca^2+^ Sensor-Kinase CBL1-CIPK5 Complex. Dev. Cell.

[127] Jagodzik P., Tajdel-Zielinska M., Ciesla A., Marczak M., Ludwikow A. (2018). Mitogen-Activated Protein Kinase Cascades in Plant Hormone Signaling. Front. Plant Sci..

[128] Meng X., Zhang S. (2013). MAPK Cascades in Plant Disease Resistance Signaling. Annu. Rev. Phytopathol..

[129] Ichimura K., Mizoguchi T., Yoshida R., Yuasa T., Shinozaki K. (2000). Various Abiotic Stresses Rapidly Activate *Arabidopsis* MAP Kinases ATMPK4 and ATMPK6. Plant J..

[130] Xu J., Zhang S. (2015). Mitogen-Activated Protein Kinase Cascades in Signaling Plant Growth and Development. Trends Plant Sci..

[131] Takahashi F., Yoshida R., Ichimura K., Mizoguchi T., Seo S., Yonezawa M., Maruyama K., Yamaguchi-Shinozaki K., Shinozaki K. (2007). The Mitogen-Activated Protein Kinase Cascade MKK3–MPK6 Is an Important Part of the Jasmonate Signal Transduction Pathway in *Arabidopsis*. Plant Cell.

[132] Tanarsuwongkul S., Fisher K.W., Mullis B.T., Negi H., Roberts J., Tomlin F., Wang Q., Stratmann J.W. (2024). Green Leaf Volatiles Co-opt Proteins Involved in Molecular Pattern Signalling in Plant Cells. Plant Cell Environ..

[133] Dombrowski J.E., Martin R.C. (2014). Green Leaf Volatiles, Fire and Nonanoic Acid Activate MAPkinases in the Model Grass Species *Lolium temulentum*. BMC Res. Notes.

[134] Dombrowski J.E., Martin R.C. (2018). Activation of MAP Kinases by Green Leaf Volatiles in Grasses. BMC Res. Notes.

[135] Thoma I., Loeffler C., Sinha A.K., Gupta M., Krischke M., Steffan B., Roitsch T., Mueller M.J. (2003). Cyclopentenone Isoprostanes Induced by Reactive Oxygen Species Trigger Defense Gene Activation and Phytoalexin Accumulation in Plants. Plant J..

[136] Seo S., Okamoto M., Seto H., Ishizuka K., Sano H., Ohashi Y. (1995). Tobacco MAP Kinase: A Possible Mediator in Wound Signal Transduction Pathways. Science.

[137] Asai T., Tena G., Plotnikova J., Willmann M.R., Chiu W.-L., Gomez-Gomez L., Boller T., Ausubel F.M., Sheen J. (2002). MAP Kinase Signalling Cascade in *Arabidopsis* Innate Immunity. Nature.

[138] Abe H., Urao T., Ito T., Seki M., Shinozaki K., Yamaguchi-Shinozaki K. (2003). *Arabidopsis* AtMYC2 (bHLH) and AtMYB2 (MYB) Function as Transcriptional Activators in Abscisic Acid Signaling. Plant Cell.

[139] Koh H., Joo H., Lim C.W., Lee S.C. (2023). Roles of the Pepper JAZ Protein CaJAZ1-03 and Its Interacting Partner RING-type E3 Ligase CaASRF1 in Regulating ABA Signaling and Drought Responses. Plant Cell Environ..

[140] Liu W., Jiang Y., Jin Y., Wang C., Yang J., Qi H. (2021). Drought-Induced ABA, H2O2 and JA Positively Regulate CmCAD Genes and Lignin Synthesis in Melon Stems. BMC Plant Biol..

[141] Kim Y.Y., Jung K.W., Yoo K.S., Jeung J.U., Shin J.S. (2011). A Stress-Responsive Caleosin-Like Protein, AtCLO4, Acts as a Negative Regulator of ABA Responses in Arabidopsis. Plant Cell Physiol..

[142] Partridge M., Murphy D.J. (2009). Roles of a Membrane-Bound Caleosin and Putative Peroxygenase in Biotic and Abiotic Stress Responses in *Arabidopsis*. Plant Physiol. Biochem..

[143] Blée E., Boachon B., Burcklen M., Le Guédard M., Hanano A., Heintz D., Ehlting J., Herrfurth C., Feussner I., Bessoule J.-J. (2014). The Reductase Activity of the Arabidopsis Caleosin RESPONSIVE TO DESSICATION20 Mediates Gibberellin-Dependent Flowering Time, Abscisic Acid Sensitivity, and Tolerance to Oxidative Stress. Plant Physiol..

[144] Zhu Z., An F., Feng Y., Li P., Xue L., A M., Jiang Z., Kim J.-M., To T.K., Li W. (2011). Derepression of Ethylene-Stabilized Transcription Factors (EIN3/EIL1) Mediates Jasmonate and Ethylene Signaling Synergy in *Arabidopsis*. Proc. Natl. Acad. Sci. USA.

[145] Kazan K. (2015). Diverse Roles of Jasmonates and Ethylene in Abiotic Stress Tolerance. Trends Plant Sci..

[146] Zhu C., Li X., Zhang M., Wang S., Jing B., Hu C., Thomas H.R., Zhou Y., Yu J., Hu Z. (2025). ERF.D2 Negatively Controls Drought Tolerance through Synergistic Regulation of Abscisic Acid and Jasmonic Acid in Tomato. Plant Biotechnol. J..

[147] Lee A., Cho K., Jang S., Rakwal R., Iwahashi H., Agrawal G.K., Shim J., Han O. (2004). Inverse Correlation between Jasmonic Acid and Salicylic Acid during Early Wound Response in Rice. Biochem. Biophys. Res. Commun..

[148] Yang B., Chen M., Wang T., Chen X., Li Y., Wang X., Zhu W., Xia L., Hu X., Tian J. (2019). A Metabolomic Strategy Revealed the Role of JA and SA Balance in *Clematis terniflora* DC. Response to UVB Radiation and Dark. Physiol. Plant..

[149] Lelarge-Trouverie C., Cohen M., Trémulot L., Van Breusegem F., Mhamdi A., Noctor G. (2023). Metabolite Modification in Oxidative Stress Responses: A Case Study of Two Defense Hormones. Free Radic. Biol. Med..

[150] Clouse S.D. (2011). Brassinosteroid Signal Transduction: From Receptor Kinase Activation to Transcriptional Networks Regulating Plant Development. Plant Cell.

[151] Nolan T.M., Vukašinović N., Liu D., Russinova E., Yin Y. (2020). Brassinosteroids: Multidimensional Regulators of Plant Growth, Development, and Stress Responses. Plant Cell.

[152] Zhang J., Chen W., Li X., Shi H., Lv M., He L., Bai W., Cheng S., Chu J., He K. (2023). Jasmonates Regulate Apical Hook Development by Repressing Brassinosteroid Biosynthesis and Signaling. Plant Physiol..

[153] Gan H., Wang S., Yang Z., Ma P. (2025). Molecular Decoding of Phytohormone Crosstalk: JA-Mediated Key Regulatory Nodes and Signal Integration. Plants.

[154] Chen Q., Sun J., Zhai Q., Zhou W., Qi L., Xu L., Wang B., Chen R., Jiang H., Qi J. (2011). The Basic Helix-Loop-Helix Transcription Factor MYC2 Directly Represses *PLETHORA* Expression during Jasmonate-Mediated Modulation of the Root Stem Cell Niche in *Arabidopsis*. Plant Cell.

[155] Grunewald W., Vanholme B., Pauwels L., Plovie E., Inzé D., Gheysen G., Goossens A. (2009). Expression of the *Arabidopsis* Jasmonate Signalling Repressor JAZ1/TIFY10A Is Stimulated by Auxin. EMBO Rep..

[156] Sun J., Xu Y., Ye S., Jiang H., Chen Q., Liu F., Zhou W., Chen R., Li X., Tietz O. (2009). *Arabidopsis ASA1* Is Important for Jasmonate-Mediated Regulation of Auxin Biosynthesis and Transport during Lateral Root Formation. Plant Cell.

[157] Hou X., Lee L.Y.C., Xia K., Yan Y., Yu H. (2010). DELLAs Modulate Jasmonate Signaling via Competitive Binding to JAZs. Dev. Cell.

[158] Yang D.-L., Yao J., Mei C.-S., Tong X.-H., Zeng L.-J., Li Q., Xiao L.-T., Sun T., Li J., Deng X.-W. (2012). Plant Hormone Jasmonate Prioritizes Defense over Growth by Interfering with Gibberellin Signaling Cascade. Proc. Natl. Acad. Sci. USA.

[159] Gorina S., Ogorodnikova A., Mukhtarova L., Toporkova Y. (2022). Gene Expression Analysis of Potato (*Solanum tuberosum* L.) Lipoxygenase Cascade and Oxylipin Signature under Abiotic Stress. Plants.

[160] De Domenico S., Bonsegna S., Horres R., Pastor V., Taurino M., Poltronieri P., Imtiaz M., Kahl G., Flors V., Winter P. (2012). Transcriptomic Analysis of Oxylipin Biosynthesis Genes and Chemical Profiling Reveal an Early Induction of Jasmonates in Chickpea Roots under Drought Stress. Plant Physiol. Biochem..

[161] Xing Q., Liao J., Cao S., Li M., Lv T., Qi H. (2020). *CmLOX10* Positively Regulates Drought Tolerance through Jasmonic Acid-Mediated Stomatal Closure in Oriental Melon (*Cucumis melo* var. *makuwa* Makino). Sci. Rep..

[162] Seo J., Joo J., Kim M., Kim Y., Nahm B.H., Song S.I., Cheong J., Lee J.S., Kim J., Choi Y.D. (2011). OsbHLH148, a Basic Helix-loop-helix Protein, Interacts with OsJAZ Proteins in a Jasmonate Signaling Pathway Leading to Drought Tolerance in Rice. Plant J..

[163] Fu J., Wu H., Ma S., Xiang D., Liu R., Xiong L. (2017). OsJAZ1 Attenuates Drought Resistance by Regulating JA and ABA Signaling in Rice. Front. Plant Sci..

[164] Zhang M., Zhao R., Huang K., Wei Z., Guo B., Huang S., Li Z., Jiang W., Wu T., Du X. (2023). OsWRKY76 Positively Regulates Drought Stress via OsbHLH148-Mediated Jasmonate Signaling in Rice. Front. Plant Sci..

[165] De Ollas C., Arbona V., Gómez-Cadenas A., Dodd I.C. (2018). Attenuated Accumulation of Jasmonates Modifies Stomatal Responses to Water Deficit. J. Exp. Bot..

[166] Savchenko T., Kolla V.A., Wang C.-Q., Nasafi Z., Hicks D.R., Phadungchob B., Chehab W.E., Brandizzi F., Froehlich J., Dehesh K. (2014). Functional Convergence of Oxylipin and Abscisic Acid Pathways Controls Stomatal Closure in Response to Drought. Plant Physiol..

[167] Canales F.J., Montilla-Bascón G., Rispail N., Arbona V., Mur L.A.J., Prats E. (2026). Differential Jasmonate Profiles in Oat Roots and Leaves Reveal a Role for 12-Oxo Phytodienoic Acid (OPDA) in Drought Tolerance by Modulating Root Growth. Plants.

[168] Grebner W., Stingl N.E., Oenel A., Mueller M.J., Berger S. (2013). Lipoxygenase6-Dependent Oxylipin Synthesis in Roots Is Required for Abiotic and Biotic Stress Resistance of *Arabidopsis*. Plant Physiol..

[169] Aubert Y., Vile D., Pervent M., Aldon D., Ranty B., Simonneau T., Vavasseur A., Galaud J.-P. (2010). RD20, a Stress-Inducible Caleosin, Participates in Stomatal Control, Transpiration and Drought Tolerance in *Arabidopsis thaliana*. Plant Cell Physiol..

[170] Shen J., Shen Y., Li Y., Sun L., Gu H., Wang S., Li X., Fan K., Chen S., Wang Y. (2026). The Green Leaf Volatile Z-3-Hexenyl Acetate Improves Drought Stress Resistance of Tea Plants via Regulating the Phenylpropanoid Biosynthesis Pathway. Sci. Hortic..

[171] Harb A., Krishnan A., Ambavaram M.M.R., Pereira A. (2010). Molecular and Physiological Analysis of Drought Stress in Arabidopsis Reveals Early Responses Leading to Acclimation in Plant Growth. Plant Physiol..

[172] Zhang H., Zhang Q., Zhai H., Li Y., Wang X., Liu Q., He S. (2017). Transcript Profile Analysis Reveals Important Roles of Jasmonic Acid Signalling Pathway in the Response of Sweet Potato to Salt Stress. Sci. Rep..

[173] Ding H., Lai J., Wu Q., Zhang S., Chen L., Dai Y.-S., Wang C., Du J., Xiao S., Yang C. (2016). Jasmonate Complements the Function of *Arabidopsis* Lipoxygenase3 in Salinity Stress Response. Plant Sci..

[174] Zhao Y., Dong W., Zhang N., Ai X., Wang M., Huang Z., Xiao L., Xia G. (2014). A Wheat Allene Oxide Cyclase Gene Enhances Salinity Tolerance via Jasmonate Signaling. Plant Physiol..

[175] Valenzuela C.E., Acevedo-Acevedo O., Miranda G.S., Vergara-Barros P., Holuigue L., Figueroa C.R., Figueroa P.M. (2016). Salt Stress Response Triggers Activation of the Jasmonate Signaling Pathway Leading to Inhibition of Cell Elongation in *Arabidopsis* Primary Root. J. Exp. Bot..

[176] Asfaw K.G., Liu Q., Eghbalian R., Purper S., Akaberi S., Dhakarey R., Münch S.W., Wehl I., Bräse S., Eiche E. (2022). The Jasmonate Biosynthesis Gene OsOPR7 Can Mitigate Salinity Induced Mitochondrial Oxidative Stress. Plant Sci..

[177] Lung S.-C., Lai S.H., Wang H., Zhang X., Liu A., Guo Z.-H., Lam H.-M., Chye M.-L. (2022). Oxylipin Signaling in Salt-Stressed Soybean Is Modulated by Ligand-Dependent Interaction of Class II Acyl-CoA-Binding Proteins with Lipoxygenase. Plant Cell.

[178] Zhu J., Wei X., Yin C., Zhou H., Yan J., He W., Yan J., Li H. (2023). ZmEREB57 Regulates OPDA Synthesis and Enhances Salt Stress Tolerance through Two Distinct Signalling Pathways in *Zea mays*. Plant Cell Environ..

[179] De Domenico S., Taurino M., Gallo A., Poltronieri P., Pastor V., Flors V., Santino A. (2019). Oxylipin Dynamics in *Medicago truncatula* in Response to Salt and Wounding Stresses. Physiol. Plant..

[180] Jing P., Kong D., Ji L., Kong L., Wang Y., Peng L., Xie G. (2021). OsClo5 Functions as a Transcriptional Co-repressor by Interacting with OsDi19-5 to Negatively Affect Salt Stress Tolerance in Rice Seedlings. Plant J..

[181] Tian S., Guo R., Zou X., Zhang X., Yu X., Zhan Y., Ci D., Wang M., Wang Y., Si T. (2019). Priming With the Green Leaf Volatile (Z)-3-Hexeny-1-Yl Acetate Enhances Salinity Stress Tolerance in Peanut (*Arachis hypogaea* L.) Seedlings. Front. Plant Sci..

[182] Pan J., Sohail H., Sharif R., Hu Q., Song J., Qi X., Chen X., Xu X. (2024). Cucumber JASMONATE ZIM-DOMAIN 8 Interaction with Transcription Factor MYB6 Impairs Waterlogging-Triggered Adventitious Rooting. Plant Physiol..

[183] Hong Y., Zhou S., Zhang J., Lv Y., Yao N., Liu X. (2025). CtMYB63 Enhances the Waterlogging Tolerance of Safflower through the JA Signalling Pathway. Plant Physiol. Biochem..

[184] Savchenko T., Rolletschek H., Heinzel N., Tikhonov K., Dehesh K. (2019). Waterlogging Tolerance Rendered by Oxylipin-Mediated Metabolic Reprogramming in *Arabidopsis*. J. Exp. Bot..

[185] Islam F., Khan M.S.S., Ahmed S., Abdullah M., Hannan F., Chen J. (2023). OsLPXC Negatively Regulates Tolerance to Cold Stress via Modulating Oxidative Stress, Antioxidant Defense and JA Accumulation in Rice. Free Radic. Biol. Med..

[186] Li W., Wen Y., Quan J., Gao M., Shang C., Liu X., Liu G., Hu X., Li J. (2025). Regulation of Jasmonic Acid Signalling in Tomato Cold Stress Response: Insights into the MYB15—LOXD and MYB15—MYC2—LOXD Regulatory Modules. Plant Biotechnol. J..

[187] Wang F., Guo Z., Li H., Wang M., Onac E., Zhou J., Xia X., Shi K., Yu J., Zhou Y. (2016). Phytochrome A and B Function Antagonistically to Regulate Cold Tolerance via Abscisic Acid-Dependent Jasmonate Signaling. Plant Physiol..

[188] Cofer T.M., Engelberth M., Engelberth J. (2018). Green Leaf Volatiles Protect Maize (*Zea mays*) Seedlings against Damage from Cold Stress. Plant Cell Environ..

[189] Yokoyama M., Kurusu T., Ohno H., Ifuku O., Harada R., Tada Y. (2025). Oxylipin KODA Enhances the Early Growth of Rice (*Oryza sativa* L.) under Low-Temperature Stress at Night to Simulate a Natural Temperature Condition. Plant Biotechnol..

[190] Tian X., Wang F., Zhao Y., Lan T., Yu K., Zhang L., Qin Z., Hu Z., Yao Y., Ni Z. (2020). Heat Shock Transcription Factor A1b Regulates Heat Tolerance in Wheat and Arabidopsis through OPR3 and Jasmonate Signalling Pathway. Plant Biotechnol. J..

[191] Monte I., Kneeshaw S., Franco-Zorrilla J.M., Chini A., Zamarreño A.M., García-Mina J.M., Solano R. (2020). An Ancient COI1-Independent Function for Reactive Electrophilic Oxylipins in Thermotolerance. Curr. Biol..

[192] Ronzan M., Piacentini D., Fattorini L., Federica D.R., Caboni E., Eiche E., Ziegler J., Hause B., Riemann M., Betti C. (2019). Auxin-Jasmonate Crosstalk in *Oryza sativa* L. Root System Formation after Cadmium and/or Arsenic Exposure. Environ. Exp. Bot..

[193] López-Orenes A., Alba J.M., Kant M.R., Calderón A.A., Ferrer M.A. (2020). OPDA and ABA Accumulation in Pb-Stressed Zygophyllum Fabago Can Be Primed by Salicylic Acid and Coincides with Organ-Specific Differences in Accumulation of Phenolics. Plant Physiol. Biochem..

[194] Mousavi S.R., Niknejad Y., Fallah H., Tari D.B. (2020). Methyl Jasmonate Alleviates Arsenic Toxicity in Rice. Plant Cell Rep..

[195] Singh I., Shah K. (2014). Exogenous Application of Methyl Jasmonate Lowers the Effect of Cadmium-Induced Oxidative Injury in Rice Seedlings. Phytochemistry.

[196] Li C., Du J., Xu H., Feng Z., Chater C.C.C., Duan Y., Yang Y., Sun X. (2024). UVR8-TCP4-LOX2 Module Regulates UV-B Tolerance in Arabidopsis. J. Integr. Plant Biol..

[197] Liu Y., Wang J., Liu X., Liao T., Ren H., Liu L., Huang X. (2025). The UV-B Photoreceptor UVR8 Interacts with the LOX1 Enzyme to Promote Stomatal Closure through the LOX-Derived Oxylipin Pathway. Plant Cell.

[198] Kılıç M., Gollan P.J., Aro E.-M., Rintamäki E. (2025). Jasmonic Acid Signaling and Glutathione Coordinate Plant Recovery from High Light Stress. Plant Physiol..

[199] Lan F., Zhang Q., Kang Y., Zhu L., Liu M., Fu A., Huang R., Fu Q., Peng Y., Wang L. (2026). The MYC2—MYB113 Module Governs JA Signaling to Regulate Anthocyanin Accumulation and Secondary Wall Thickening under High Light in *Populus*. New Phytol..

[200] Savchenko T., Yanykin D., Khorobrykh A., Terentyev V., Klimov V., Dehesh K. (2017). The Hydroperoxide Lyase Branch of the Oxylipin Pathway Protects against Photoinhibition of Photosynthesis. Planta.

[201] Jin J., Zhao M., Jing T., Wang J., Lu M., Pan Y., Du W., Zhao C., Bao Z., Zhao W. (2023). (*Z*)-3-Hexenol Integrates Drought and Cold Stress Signaling by Activating Abscisic Acid Glucosylation in Tea Plants. Plant Physiol..

[202] Balfagón D., Sengupta S., Gómez-Cadenas A., Fritschi F.B., Azad R.K., Mittler R., Zandalinas S.I. (2019). Jasmonic Acid Is Required for Plant Acclimation to a Combination of High Light and Heat Stress. Plant Physiol..

[203] Rahman M.M., Mostofa M.G., Keya S.S., Ghosh P.K., Abdelrahman M., Anik T.R., Gupta A., Tran L.-S.P. (2024). Jasmonic Acid Priming Augments Antioxidant Defense and Photosynthesis in Soybean to Alleviate Combined Heat and Drought Stress Effects. Plant Physiol. Biochem..

[204] Oshita T., Sim J., Anee T.I., Kiyono H., Nozu C., Suzuki N. (2023). Attenuation of Negative Effects Caused by a Combination of Heat and Cadmium Stress in *Arabidopsis thaliana* Deficient in Jasmonic Acid Synthesis. J. Plant Physiol..

[205] Parihar P., Singh R., Singh A., Prasad S.M. (2021). Role of Oxylipin on Luffa Seedlings Exposed to NaCl and UV-B Stresses: An Insight into Mechanism. Plant Physiol. Biochem..

[206] Kamal A.H.M., Komatsu S. (2016). Jasmonic Acid Induced Protein Response to Biophoton Emissions and Flooding Stress in Soybean. J. Proteom..

[207] Xu L., Pan R., Zhou M., Xu Y., Zhang W. (2019). Lipid Remodelling Plays an Important Role in Wheat (*Triticum aestivum*) Hypoxia Stress. Funct. Plant Biol..

[208] Xie L.-J., Zhou Y., Chen Q.-F., Xiao S. (2021). New Insights into the Role of Lipids in Plant Hypoxia Responses. Prog. Lipid Res..

[209] Agrawal G.K., Jwa N.-S., Shibato J., Han O., Iwahashi H., Rakwal R. (2003). Diverse Environmental Cues Transiently Regulate OsOPR1 of the “Octadecanoid Pathway” Revealing Its Importance in Rice Defense/Stress and Development. Biochem. Biophys. Res. Commun..

[210] Lee S.H., Ahn S.J., Im Y.J., Cho K., Chung G.-C., Cho B.-H., Han O. (2005). Differential Impact of Low Temperature on Fatty Acid Unsaturation and Lipoxygenase Activity in Figleaf Gourd and Cucumber Roots. Biochem. Biophys. Res. Commun..

[211] Copolovici L., Kännaste A., Pazouki L., Niinemets Ü. (2012). Emissions of Green Leaf Volatiles and Terpenoids from *Solanum lycopersicum* Are Quantitatively Related to the Severity of Cold and Heat Shock Treatments. J. Plant Physiol..

[212] Wu Z., Guo Z., Wang K., Wang R., Fang C. (2023). Comparative Metabolomic Analysis Reveals the Role of OsHPL1 in the Cold-Induced Metabolic Changes in Rice. Plants.

[213] Chaudhary R., Baliyan S., Khungar L., Sirohi P., Singh S., Mishra S.K., Rajkumar M.S., Saini S.S., Germain H., Sircar D. (2026). Overexpression of Barley Heat Stress Transcription Factor HvHsfA6a Provides Thermotolerance by Modulating Transcriptome and Metabolome. Plant J..

[214] Rakwal R., Agrawal G.K., Yonekura M. (1999). Separation of Proteins from Stressed Rice (*Oryza sativa* L.) Leaf Tissues by Two-Dimensional Polyacrylamide Gel Electrophoresis: Induction of Pathogenesis-Related and Cellular Protectant Proteins by Jasmonic Acid, UV Irradiation and Copper Chloride. Electrophoresis.

[215] Wang H., Hao J., Chen X., Hao Z., Wang X., Lou Y., Peng Y., Guo Z. (2007). Overexpression of Rice WRKY89 Enhances Ultraviolet B Tolerance and Disease Resistance in Rice Plants. Plant Mol. Biol..

[216] Xu W., Wang T., Xu S., Li F., Deng C., Wu L., Wu Y., Bian P. (2016). UV-C-Induced Alleviation of Transcriptional Gene Silencing through Plant–Plant Communication: Key Roles of Jasmonic Acid and Salicylic Acid Pathways. Mutat. Res.-Fundam. Mol. Mech. Mutagen..

[217] Quan J., Song S., Abdulrashid K., Chai Y., Yue M., Liu X. (2018). Separate and Combined Response to UV-B Radiation and Jasmonic Acid on Photosynthesis and Growth Characteristics of Scutellaria Baicalensis. Int. J. Mol. Sci..

[218] Müller S.M., Wang S., Telman W., Liebthal M., Schnitzer H., Viehhauser A., Sticht C., Delatorre C., Wirtz M., Hell R. (2017). The Redox-sensitive Module of Cyclophilin 20-3, 2-cysteine Peroxiredoxin and Cysteine Synthase Integrates Sulfur Metabolism and Oxylipin Signaling in the High Light Acclimation Response. Plant J..

[219] Luo Y., Teng S., Yin H., Zhang S., Tuo X., Tran L.-S.P. (2021). Transcriptome Analysis Reveals Roles of Anthocyanin- and Jasmonic Acid-Biosynthetic Pathways in Rapeseed in Response to High Light Stress. Int. J. Mol. Sci..

[220] Gonzalez Ibarra A.A., Wrobel K., Yanez Barrientos E., Corrales Escobosa A.R., Gutierrez Corona J.F., Enciso Donis I., Wrobel K. (2019). Impact of Cr(VI) on the Oxidation of Polyunsaturated Fatty Acids in *Helianthus annuus* Roots Studied by Metabolomic Tools. Chemosphere.

[221] Maksymiec W., Wianowska D., Dawidowicz A.L., Radkiewicz S., Mardarowicz M., Krupa Z. (2005). The Level of Jasmonic Acid in *Arabidopsis thaliana* and *Phaseolus coccineus* Plants under Heavy Metal Stress. J. Plant Physiol..

[222] Gajewska E., Witusińska A., Bernat P. (2024). Nickel-Induced Oxidative Stress and Phospholipid Remodeling in Cucumber Leaves. Plant Sci..

[223] Ali M., Kumar D., Tikoria R., Sharma R., Parkirti P., Vikram V., Kaushal K., Ohri P. (2023). Exploring the Potential Role of Hydrogen Sulfide and Jasmonic Acid in Plants during Heavy Metal Stress. Nitric Oxide.

[224] Kaushik S., Ranjan A., Sidhu A., Singh A.K., Sirhindi G. (2024). Cadmium Toxicity: Its’ Uptake and Retaliation by Plant Defence System and Ja Signaling. Biometals.

